# Machine-learning algorithms define pathogen-specific local immune fingerprints in peritoneal dialysis patients with bacterial infections

**DOI:** 10.1016/j.kint.2017.01.017

**Published:** 2017-07

**Authors:** Jingjing Zhang, Ida M. Friberg, Ann Kift-Morgan, Gita Parekh, Matt P. Morgan, Anna Rita Liuzzi, Chan-Yu Lin, Kieron L. Donovan, Chantal S. Colmont, Peter H. Morgan, Paul Davis, Ian Weeks, Donald J. Fraser, Nicholas Topley, Matthias Eberl

**Affiliations:** 1 Division of Infection and Immunity, School of Medicine, Cardiff University, Cardiff, UK; 2 Mologic Ltd., Bedford Technology Park, Thurleigh, Bedford, UK; 3 Directorate of Critical Care, Cardiff and Vale University Health Board, University Hospital of Wales, Heath Park, Cardiff, UK; 4 Kidney Research Center, Chang Gung Memorial Hospital, Chang Gung University, College of Medicine, Taoyuan City, Taiwan; 5 Wales Kidney Research Unit, Heath Park Campus, Cardiff, UK; 6 Directorate of Nephrology and Transplantation, Cardiff and Vale University Health Board, University Hospital of Wales, Heath Park, Cardiff, UK; 7 Cardiff Business School, Cardiff University, Cardiff, UK; 8 Systems Immunity Research Institute, Cardiff University, Cardiff, UK

**Keywords:** biomarkers, inflammation, machine learning methods, microbial infection, peritoneal dialysis

## Abstract

The immune system has evolved to sense invading pathogens, control infection, and restore tissue integrity. Despite symptomatic variability in patients, unequivocal evidence that an individual's immune system distinguishes between different organisms and mounts an appropriate response is lacking. We here used a systematic approach to characterize responses to microbiologically well-defined infection in a total of 83 peritoneal dialysis patients on the day of presentation with acute peritonitis. A broad range of cellular and soluble parameters was determined in peritoneal effluents, covering the majority of local immune cells, inflammatory and regulatory cytokines and chemokines as well as tissue damage–related factors. Our analyses, utilizing machine-learning algorithms, demonstrate that different groups of bacteria induce qualitatively distinct local immune fingerprints, with specific biomarker signatures associated with Gram-negative and Gram-positive organisms, and with culture-negative episodes of unclear etiology. Even more, within the Gram-positive group, unique immune biomarker combinations identified streptococcal and non-streptococcal species including coagulase-negative *Staphylococcus* spp. These findings have diagnostic and prognostic implications by informing patient management and treatment choice at the point of care. Thus, our data establish the power of non-linear mathematical models to analyze complex biomedical datasets and highlight key pathways involved in pathogen-specific immune responses.

The immune system is an intricate network of specialized cell types and molecular structures evolved to sense and target invading pathogens, control and clear the infection, and repair and restore the integrity of affected tissues and organs. The human body is constantly exposed to a plethora of pathogenic, opportunistic, commensal, and environmental microorganisms and has developed mechanisms to discriminate between harmful and harmless colonization through receptors and pathways that specifically recognize pathogen and danger-associated molecular patterns and unique antigenic epitopes.^1^, ^2^, ^3^, ^4^ However, unequivocal evidence that the human immune system distinguishes between different types of organisms in a physiologic context and mounts appropriate responses that are distinct enough to be exploited as rapid diagnostic indicators driving appropriate therapy is lacking.^5^, ^6^, ^7^, ^8^, ^9^, ^10^

Individuals with end-stage kidney disease receiving peritoneal dialysis (PD) serve as well-defined exemplar of a clinical infection requiring immediate medical intervention. Peritonitis is a common complication of PD and remains a major cause of early dropout, technical failure, and mortality.^11^, ^12^ In addition to its clinical relevance for individuals with end-stage kidney failure who depend on dialysis as life-saving renal replacement therapy, PD offers unparalleled insights into complex local cell interactions and molecular mechanisms that underpin the clinical severity of infectious episodes and that are readily translatable to improve patient management and outcomes.^13^, ^14^, ^15^ Importantly, peritoneal effluent can be sampled repeatedly and noninvasively, thus providing early and continuous access to the site of infection, even before antibiotic treatment is initiated. Moreover, PD-related peritonitis is caused by a wide spectrum of bacterial species, thereby allowing the study of acute responses to defined groups of organisms under closely related conditions.^6^, ^15^ However, although highly elevated white cell counts with a proportion of >50% granulocytes in the peritoneal effluent are used as indicators of peritonitis, only little progress has been made with regard to reliable discrimination between infection and noninfectious inflammation. Culture-based diagnosis of infection is slow and unsatisfactory, and rapid identification of disease-causing organisms using molecular techniques with sufficient sensitivity and specificity remains a challenge.^11^, ^12^, ^16^ Treatment of peritonitis therefore continues to be largely empirical, and early but untargeted treatment with broad-spectrum antibiotics and antifungals is recommended.^12^, ^17^

As alternative to organism-based diagnostics, we aimed at exploiting the human host response and used a systematic approach based on machine learning algorithms to identify diagnostically relevant, pathogen-specific local immune fingerprints in PD patients who presented with acute peritonitis. The introduction of “big data” technologies in biomedical sciences to address the complexity of the molecular and cellular mechanisms underlying disease has brought about an increasing need for advanced statistical models, machine learning, and pattern recognition techniques. In particular, wrapped feature selection methods have proved highly efficient for finding the best feature combination compared with time-consuming exhaustive searches.^18^ Support Vector Machines (SVMs) are data-driven methods that try to find a separating hyperplane with the maximal “margin” for classification problems and that can also be used for regression or density estimation.^19^, ^20^, ^21^ Artificial neural networks (ANNs) are inspired by biological neural networks with data processing from the input through a network of multiple nodes that are connected with each other in different layers.^22^, ^23^, ^24^ Random Forests (RFs) are ensemble methods constructed on multiple decision trees for classification and regression.^25^, ^26^, ^27^ By combining biomarker measurements during acute peritonitis and feature selection approaches based on SVMs, ANNs, and RFs, our findings demonstrate the power of advanced mathematical models to analyze complex biomedical datasets and highlight key pathways involved in pathogen-specific inflammatory responses at the site of infection. The observation that different infecting bacteria induce consistent and unique local immune responses has immediate diagnostic implications at the point of care by directing appropriate antibiotic treatment before conventional microbiological culture results become available.

## Results

### Local immune biomarkers form distinct hierarchical clusters

**Figure 1:**
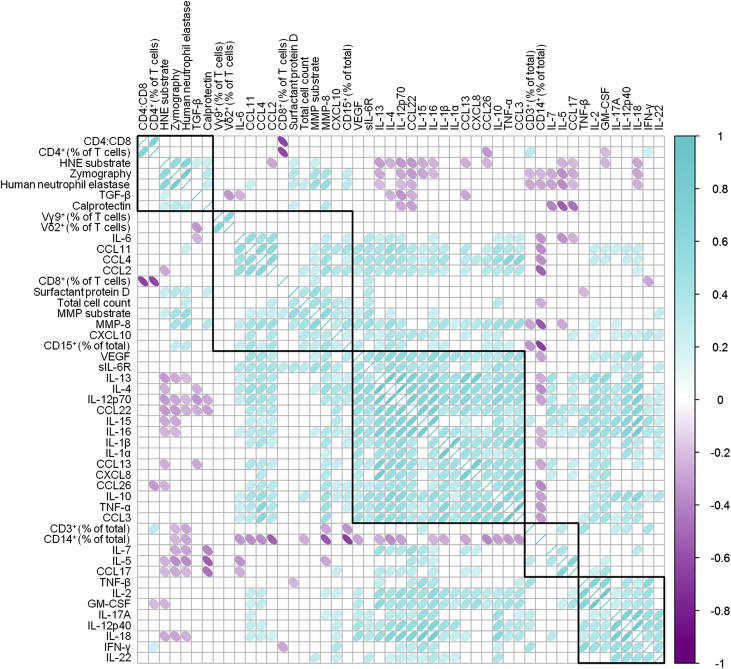
**Correlation analysis of local biomarkers in a total of 83 peritoneal dialysis patients on the day of presentation with acute peritonitis.** Ellipses depict the correlation coefficients for each pair of biomarkers in the corresponding cell of the matrix, with the direction of the dip and the color of the shading representing positive and negative correlations, respectively. Only pairs with significant correlations (*P* < 0.05) are shown. Analyses were performed using the corrplot R and Hmisc R packages. GM-CSF, granulocyte macrophage colony-stimulating factor; HNE, human neutrophil elastase; IFN-γ, interferon-γ; IL, interleukin; MMP, matrix metalloproteinase; sIL-6R, soluble IL-6 receptor; TGF-β, transforming growth factor-β; TNF-α, tumor necrosis factor-α; VEGF, vascular endothelial growth factor.

In order to define combinations of local biomarkers that would constitute relevant disease-specific immune fingerprints, we measured a broad range of cellular and soluble biomarkers in 83 PD patients presenting with microbiologically well-defined episodes of acute peritonitis (Table 1). To cover the breadth and the complexity of local inflammatory and regulatory immune responses during early infection, these biomarkers included frequencies and total numbers of infiltrating leukocytes as well as levels of common cytokines, chemokines, and tissue damage–associated molecules, the majority of which were elevated during acute peritonitis compared with baseline parameters in stable individuals (Supplementary Table S1). Perhaps not surprisingly, due to the redundant roles of many inflammatory mediators within the human immune system, some of the 49 biomarkers correlated with each other and formed 5 distinct hierarchical clusters during acute peritonitis (Figure 1). These data suggested that a signature comprising as few as 5 parameters might already suffice to define a reliable immune fingerprint.

**Table 1:** Characteristics of the patient cohort investigated in this study

	Stable		No growth		Gram-positive		Gram-negative	
	no.	%	no.	%	no.	%	no.	%
Age, yr, mean ± SEM	70.1 ± 2.2		63.8 ± 2.9		65.9 ± 2.1		68.6 ± 3.3	
18–40	1	4.2	1	5.3	4	8.5	1	5.9
40–50	1	4.2	1	5.3	3	6.4	0	0
50–60	0	0	1	5.3	6	12.8	3	17.7
60–70	8	33.3	11	57.9	14	29.8	3	17.7
70–80	10	41.7	3	15.8	12	25.5	8	47.1
≥80	4	16.7	2	10.5	8	17.0	2	11.8
Sex								
Male	18	75	11	57.9	32	68.1	9	52.9
Female	6	25	8	42.1	15	31.9	8	47.1
Days on PD, mean ± SEM	1165.1 ± 186.5		638.8 ± 163.5		1022.9 ± 144.2		768.5 ± 245.8	
0–30	0	0	3	15.8	1	2.1	0	0
30–360	1	4.2	4	21.1	15	31.9	9	52.9
360–720	9	37.5	6	31.6	8	17.0	3	17.7
720–1800	8	33.3	4	21.1	16	34.0	3	17.7
1800–3600	5	20.8	2	10.5	6	12.8	1	5.9
≥3600	1	4.2	0	0	1	2.1	1	5.9
Hypertension								
Yes	12	50	2	10.5	14	29.8	7	41.2
No	12	50	17	89.5	33	70.2	10	58.8
Diabetes								
Yes	7	29.2	6	31.6	13	27.7	7	41.2
No	17	70.8	13	68.4	34	72.3	10	58.8
Technique failure								
Days 0–14	N/A		1	5.3	2	4.3	2	11.8
Days 14–30	N/A		2	10.5	3	6.5	2	11.8
Days 30–90	N/A		1	5.3	6	12.9	4	23.5
Mortality								
Days 0–14	N/A		1	5.3	0	0	1	5.9
Days 14–90	N/A		1	5.3	0	0	1	5.9

N/A, not available; PD, peritoneal dialysis.

### Feature selection methods define local fingerprints associated with Gram-negative infections

**Figure 2:**
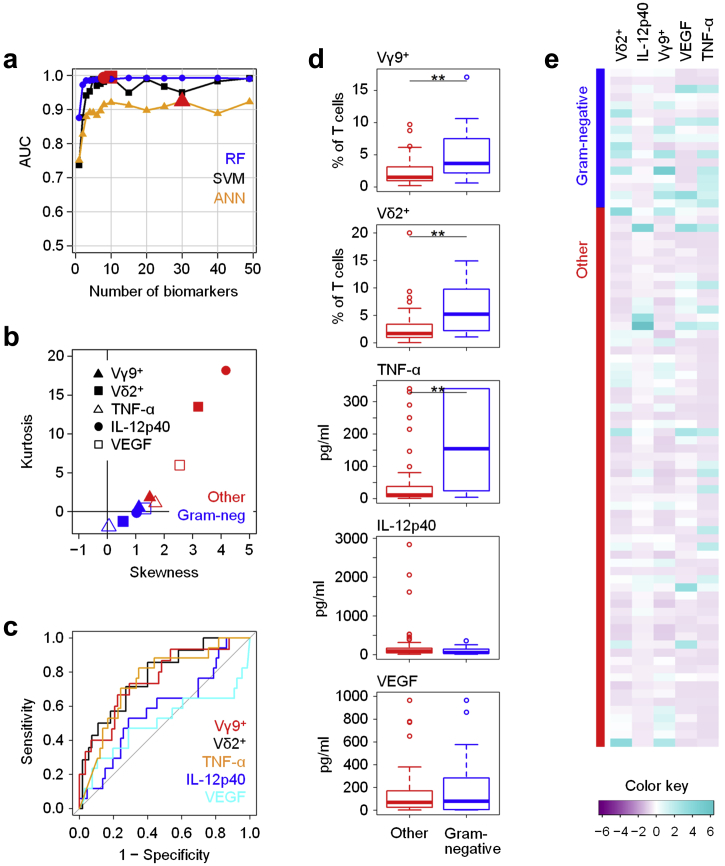
**Identification of local immune fingerprints associated with peritonitis caused by Gram-negative bacteria.** (**a**) Performance of recursive feature elimination models based on Random Forest (RF), Support Vector Machines (SVM), and artificial neural networks (ANN) for the prediction of Gram-negative infections (*N* = 17) against all other episodes of peritonitis (*N* = 66), shown as area under the receiver operating characteristic curve (AUC) depending on the number of biomarkers. Red symbols depict the maximum AUC achieved for each model. (**b**) Kurtosis and skewness of the top 5 biomarkers selected by RF-based feature elimination. (**c**) Receiver operating characteristic analysis showing specificity and sensitivity of the top 5 biomarkers. (**d**) Tukey plots of the top 5 biomarkers in patients with confirmed Gram-negative infections and with all other episodes of peritonitis, as assessed by Mann-Whitney tests (**P < 0.01). (**e**) Heat map showing the top 5 biomarkers across all patients presenting with acute peritonitis. IL, interleukin; TNF-α, tumor necrosis factor-α; VEGF, vascular endothelial growth factor.

We next divided the patients into groups according to the type of infecting organism. We initially attempted to define immune fingerprints that would reliably discriminate patients presenting with Gram-negative infections against all other cases of peritonitis (Supplementary Table S2A), based on our earlier observation of certain differences between Gram-negative and Gram-positive infections using logistic regression analyses.^6^ To this end, recursive feature elimination was used by evaluating the model performance according to the area under the receiver operating characteristic curve achieved and eliminating the least important features in each step. To reduce variability, 5 rounds of resampling methods were applied in the outer layer of the iteration, and cross-validation was used to avoid overfitting. These steps clearly demonstrated that Gram-negative infections were associated with unique different immune fingerprints. Figure 2a shows the number of features changing during feature elimination and the corresponding performance based on 3 different models, using SVMs, ANNs, and RFs. Whilst all 3 models successfully discriminated between Gram-negative infections and all other causes of peritonitis, RF-based feature elimination showed the best average performance, with the optimum biomarker combination comprising 8 features (area under receiver operating characteristic curve [AUC] = 0.993; sensitivity = 98.5% and specificity = 92.6%). In comparison, SVMs and ANNs were far less powerful for the recursive elimination of pathogen-related biomarkers, reaching overall lower degrees of sensitivity and specificity and requiring combinations comprising 10 and 30 features, respectively (Figure 2a).

The top 5 and 10 individual biomarkers selected by the 3 different models and the corresponding average performance of the models based on combinations of these biomarkers are listed in Supplementary Table S2B. Of note, although the 3 models yielded different sets of biomarkers, the frequencies of Vγ9^+^ and Vδ2^+^ T cells within peritoneal T cells featured prominently in each. These findings appear to concur with our previous data suggesting a key role for Vγ9/Vδ2 T cells in Gram-negative infections and emphasize the diagnostic potential of those cells at the point of care.^15^, ^28^ Soluble biomarkers of particular interest for the prediction of Gram-negative infections using the RF model included local levels of tumor necrosis factor (TNF)-α, interleukin (IL)-12p40, and vascular endothelial growth factor (VEGF). Taken together, our findings demonstrate that RF models showed striking performance at combinations of 5 and fewer biomarkers, thereby making it the algorithm of choice for a clinically viable prediction of the causative pathogen in individuals presenting with PD-related peritonitis.

### Internal validation of individual biomarkers constituting Gram-negative immune fingerprints

We next sought to validate the findings from the feature elimination process by assessing the distribution and performance of the top 5 biomarkers that had been selected by the RF model. Using skewness as a measure of symmetry and kurtosis as a measure of peakedness, all 5 biomarkers showed very low positive skewness and only limited or even negative kurtosis in patients presenting with Gram-negative infections (Figure 2b). In contrast, these 5 biomarkers had generally higher skewness and considerable positive kurtosis in patients with other episodes of peritonitis, especially in the case of Vδ2^+^ T-cell frequencies and levels of IL-12p40 and VEGF. Among the top 5 biomarkers, the local frequencies of Vγ9^+^ and Vδ2^+^ T cells and the levels of TNF-α on their own already showed relatively good sensitivities and specificities to identify Gram-negative infections with AUCs ≥0.75 for each biomarker, much more so than levels of IL-12p40 and VEGF (Figure 2c), but far lower than the full signature that reached an AUC of 0.99 (Supplementary Table S2B). This conclusion was supported by classic analyses using the Mann-Whitney *U* test showing that levels of Vγ9^+^ and Vδ2^+^ T cells and TNF-α, but not of IL-12p40 and VEGF, were markedly different between patients with Gram-negative infections and patients with other episodes of peritonitis (Figure 2d). These results confirmed the importance of the biomarkers selected by recursive feature elimination, especially of local levels of Vγ9^+^ and Vδ2^+^ T cells and of TNF-α, in identifying Gram-negative organisms. However, our analyses also identified shortcomings of conventional statistical methods that were especially apparent when visualizing the individual readings across all patients in the form of a heat map, where the overall differences between Gram-negative and other episodes of peritonitis were not very pronounced (Figure 2e). Overall, the performances of the individual biomarkers lagged behind their combined performance in an RF model, demonstrating the importance of defining complex signatures comprising distinct biomarkers and of assessing their relationships in nonlinear models.

### Patients with culture-negative episodes of peritonitis show distinct local immune fingerprints associated with milder inflammation

**Figure 3:**
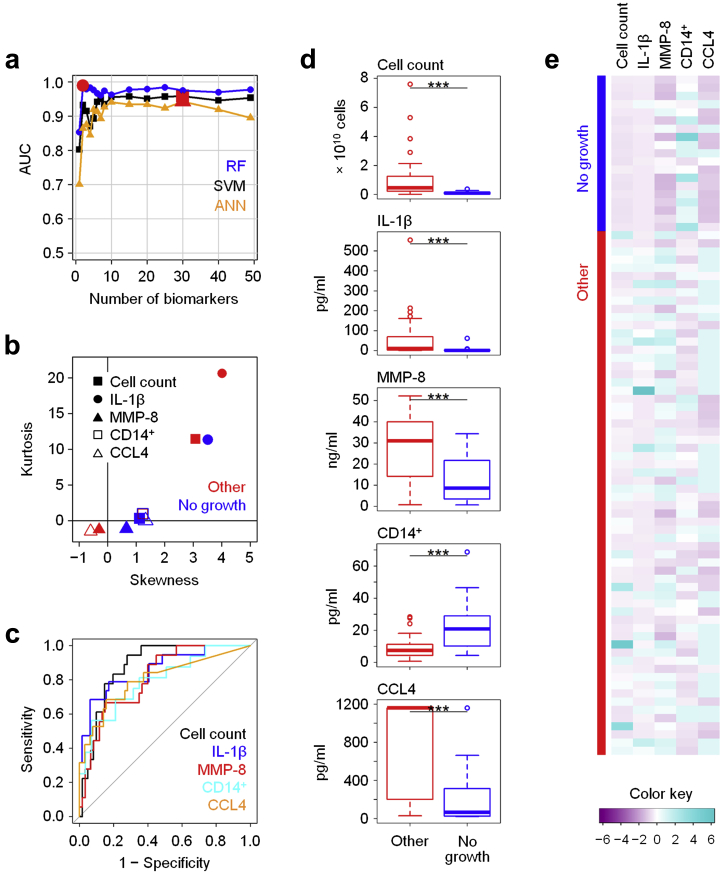
**Local immune fingerprints in culture-negative episodes of peritonitis.** (**a**) Performance of Random Forest (RF), Support Vector Machine (SVM), and artificial neural network (ANN)–based feature elimination models for the prediction of culture-negative episodes (no growth, *N* = 19) against microbiologically confirmed infections (other, *N* = 64), shown as area under the curve (AUC) depending on the number of biomarkers. Red symbols depict the maximum AUC for each model. (**b**) Kurtosis and skewness of the top 5 biomarkers selected by RF-based feature elimination. (**c**) Receiver operating characteristic analysis showing specificity and sensitivity of the top 5 biomarkers. (**d**) Tukey plots of the top 5 biomarkers in patients with culture-negative peritonitis and with infectious (other) episodes of peritonitis, as assessed by Mann-Whitney tests (***P < 0.001). (**e**) Heat map showing the top 5 biomarkers across all patients presenting with acute peritonitis. IL, interleukin; MMP, matrix metalloproteinase.

Although microbiological culture remains the method of choice for diagnosis of infection, a considerable proportion of samples does not yield any culture results, thereby not allowing a reliable designation of the underlying cause of the inflammatory episode, which may or may not be infectious.^29^ Patients with culture-negative episodes often show less severe inflammation and have better clinical outcomes, suggesting that local biomarkers might aid in the diagnosis, treatment, and prognosis of such episodes. Here, RF models selected biomarker signatures that reliably distinguished samples with no growth from cases with confirmed bacterial infection (Figure 3a, Supplementary Tables S3A and S3B). The top 5 biomarkers all showed great potential in identifying culture-negative episodes that were characterized by relatively low total cell counts, an increased proportion of CD14^+^ monocytes/macrophages in the cellular infiltrate, and lower levels of IL-1β, matrix metalloproteinase (MMP)-8 and the chemokine CCL4 compared with confirmed infections (Figure 3b–e, Supplementary Table S3B). Despite the heterogeneity of this patient group, in which the failure to grow organisms might be due to inappropriate sampling, poor handling and culture techniques, low organism numbers, ongoing treatment with antibiotics for unrelated infections, or nonmicrobial disease such as sterile inflammation or viral infection, our findings indicate that culture-negative episodes are immunologically distinct from confirmed cases of bacterial peritonitis and are characterized by a less severe inflammatory response.

### Different types of Gram-positive bacteria induce distinct immune responses that allow discrimination between organism subgroups

We next sought to define immune fingerprints in PD patients with confirmed infections caused by Gram-positive bacteria (Supplementary Table S4A). Here, feature elimination models were able to discriminate between Gram-positive infections and other episodes, yet required combinations of ≥30 biomarkers for optimal performance and failed to reach satisfactory AUCs (Supplementary Figure S1). The best 5 biomarkers together only reached an AUC of 0.711 in the RF model, and none of the individual markers on their own—IL-17A, IL-12p40, interferon-γ, IL-1β, and total cell count—exceeded an AUC of 0.72 or stayed well below that value (Supplementary Table S4B).

**Figure 4:**
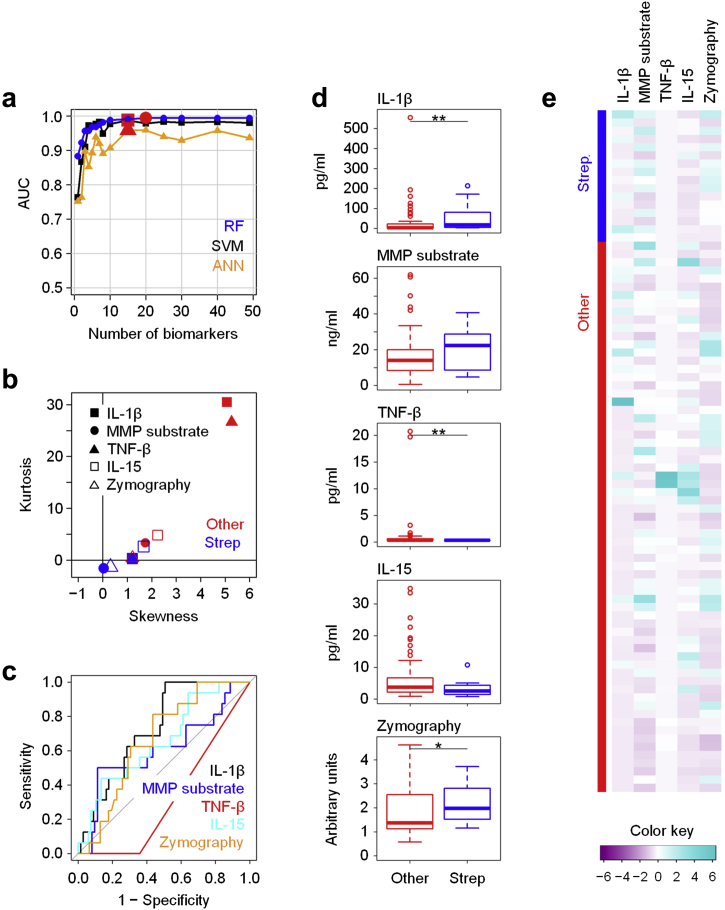
**Local immune fingerprints in streptococcal (Strep) infections.** (**a**) Performance of Random Forest (RF), Support Vector Machine (SVM), and artificial neural network (ANN)–based feature elimination models for the prediction of infections caused by streptococcal species (*Streptococcus* spp. and *Enterococcus* spp., *N* = 16) against all other episodes of peritonitis (*N* = 67), shown as area under the curve (AUC) depending on the number of biomarkers. One episode of peritonitis classified as streptococcal infection was a coinfection caused by *Enterococcus* sp. with light growth of coagulase-negative *Staphylococcus* spp. Red symbols depict the maximum AUC for each model. (**b**) Kurtosis and skewness of the top 5 biomarkers selected by RF-based feature elimination. (**c**) Receiver operating characteristic analysis showing specificity and sensitivity of the top 5 biomarkers. (**d**) Tukey plots of the top 5 biomarkers in patients with confirmed streptococcal infections and with all other episodes of peritonitis, as assessed by Mann-Whitney tests (*P < 0.05; **P < 0.01). (**e**) Heat map showing the top 5 biomarkers across all patients presenting with acute peritonitis. IL, interleukin; MMP, matrix metalloproteinase; TNF-β, tumor necrosis factor-β.

Of note, there is considerable heterogeneity in the Gram-positive organisms causing PD-related peritonitis, comprising streptococci, staphylococci, coryneforms, and other bacteria that cause clinically distinct diseases with different outcomes and require different antibiotics.^12^ We therefore attempted to define the pathogen-specific immune responses to subtypes of Gram-positive organisms. These analyses demonstrated that in the Gram-positive group, streptococcal infections caused by *Streptococcus* and *Enterococcus* species were associated with markedly distinct immune responses compared with all other cases of peritonitis (Figure 4a, Supplementary Table S5A). The most promising biomarker combination consisted of the local levels of IL-1β, TNF-β, and IL-15 together with the enzymatic activity of MMPs in the PD effluent, as measured by specific cleavage of a fluorogenic MMP substrate and by gelatin zymography on gel electrophoretic separation (Figure 4b). Individually, IL-1β, TNF-β, and zymography showed differences between streptococcal infections and all other patients, yet only an RF-based model revealed their full diagnostic potential with an AUC of 0.969 (Figure 4c–e, Supplementary Table S5B).

Similar to the definition of *Streptococcus*/*Enterococcus*-specific immune fingerprints, nonstreptococcal Gram-positive infections caused by *Staphylococcus aureus*, coagulase-negative *Staphylococcus* spp. (CNS) and *Corynebacterium* spp. were associated with biomarker signatures that distinguished them from all other episodes of peritonitis (Supplementary Figure S2, Supplementary Table S6A). Yet, despite a statistically significant difference between such nonstreptococcal Gram-positive infections and other episodes, especially for local levels of IL-17A, interferon-γ, and IL-15, RF-based algorithms were not as powerful in this case as for the above predictions of Gram-negative or streptococcal infections, most likely due to the remaining heterogeneity of the organisms in that patient group (Supplementary Table S6B).

**Figure 5:**
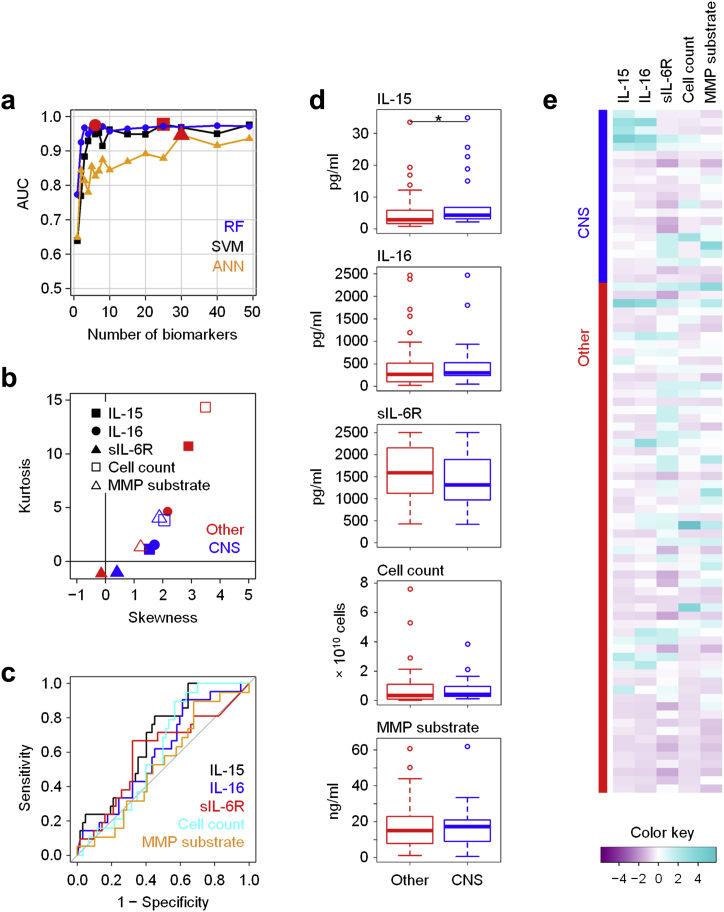
**Local immune fingerprints in coagulase-negative *Staphylococcus* (CNS) infections.** (**a**) Performance of Random Forest (RF), Support Vector Machine (SVM), and artificial neural network (ANN)–based feature elimination models for the prediction of infections caused by CNS (*Staphylococcus epidermidis* and related species; *N* = 21) against all other episodes of peritonitis (*N* = 62), shown as area under the curve (AUC) depending on the number of biomarkers. Red symbols depict the maximum AUC for each model. (**b**) Kurtosis and skewness of the top 5 biomarkers selected by RF-based feature elimination. (**c**) Receiver operating characteristic analysis showing specificity and sensitivity of the top 5 biomarkers. (**d**) Tukey plots of the top 5 biomarkers in patients with confirmed CNS infections and with all other episodes of peritonitis, as assessed by Mann-Whitney tests (*P < 0.05). (**e**) Heat map showing the top 5 biomarkers across all patients presenting with acute peritonitis. IL, interleukin; MMP, matrix metalloproteinase; sIL-6R, soluble IL-6 receptor.

Given that CNS species such as *Staphylococcus epidermidis* are the major cause of peritonitis in PD patients and are also clinically associated with a relatively benign outcome,^30^ we finally determined immune fingerprints that would specifically define CNS infections. Despite having only marginal or no statistical significance as individual biomarkers on conventional tests, the combination of IL-15, IL-16, and soluble IL-6 receptor (sIL-6R) levels, total cell count, and MMP substrate turnover showed excellent performance in the RF model, with an AUC of 0.961 (Figure 5, Supplementary Tables S7A+B), demonstrating that CNS infections are sufficiently immunologically distinct to allow a pathogen-specific diagnosis in PD patients.

### Local biomarkers on the day of presentation correlate with subsequent clinical outcomes over the following 90 days

**Figure 6:**
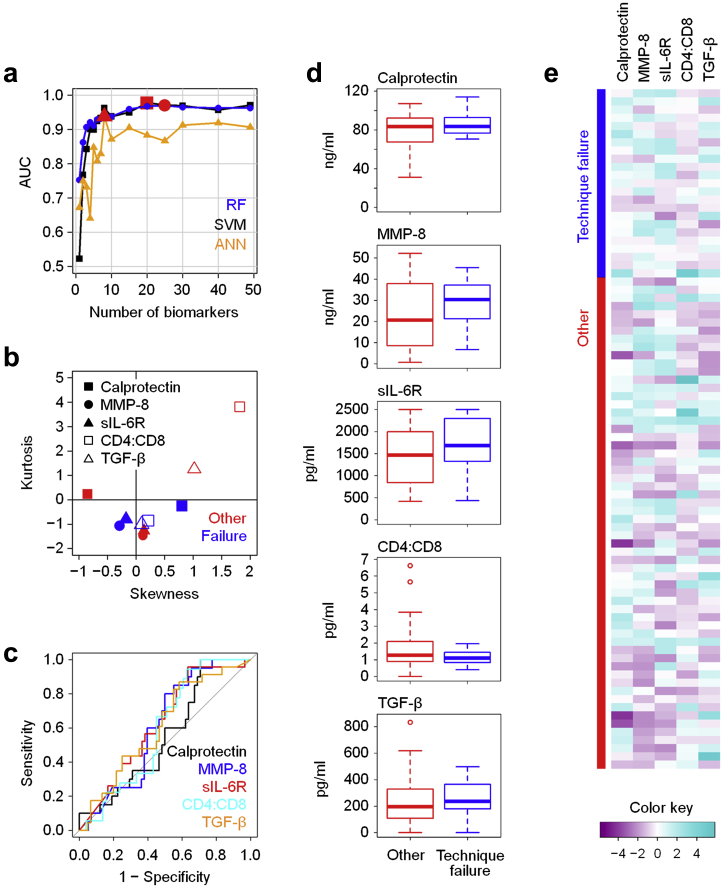
**Local immune fingerprints associated with poor clinical outcomes.** (**a**) Performance of Random Forest (RF), Support Vector Machine (SVM), and artificial neural network (ANN)–based feature elimination models for the prediction of technique failure over the next 90 days (catheter removal, transfer to hemodialysis, or peritonitis-related death; *N* = 23) against all other episodes of peritonitis (*N* = 60), shown as area under the curve (AUC) depending on the number of biomarkers. Red symbols depict the maximum AUC for each model. (**b**) Kurtosis and skewness of the top 5 biomarkers selected by RF-based feature elimination. (**c**) Receiver operating characteristic analysis showing specificity and sensitivity of the top 5 biomarkers. (**d**) Tukey plots of the top 5 biomarkers in patients with subsequent technique failure and all other patients, as assessed by Mann-Whitney tests. (**e**) Heat map showing the top 5 biomarkers across all patients presenting with acute peritonitis. MMP, matrix metalloproteinase; sIL-6R, soluble IL-6 receptor; TGF-β, transforming growth factor-β.

The nature of the causative pathogen and the underlying inflammatory response profoundly affect clinical outcomes of peritonitis in PD patients,^6^, ^15^ indicating a need for prognostic biomarkers in early disease. When applying recursive feature elimination methods to our dataset, certain models accurately predicted the risk of downstream complications, defined as technique failure over the following 90 days, including catheter removal, transfer to hemodialysis, and death (Figure 6). Patients experiencing technique failure after an episode of acute peritonitis had marginally higher levels of calprotectin, MMP-8, sIL-6R, and transforming growth factor-β in their dialysis effluent as well as lower CD4^+^ : CD8^+^ T-cell ratios compared with uncomplicated cases. Although not being significantly different on their own, the combination of these 5 parameters in RF models yielded an AUC of 0.911 with excellent sensitivity (Supplementary Tables S8A and S8B), implying that the development of prognostic tests is feasible.

**Figure 7:**
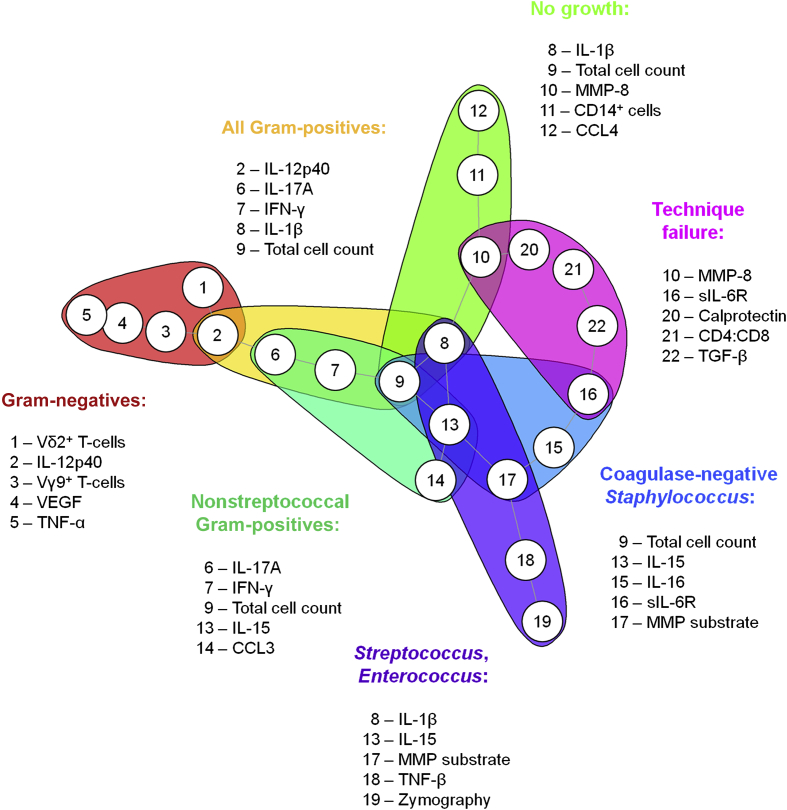
**Summary of disease-specific immune fingerprints in patients presenting with acute peritonitis.** Shown are the top 5 biomarkers associated with the type of causative organism as indicated or with the risk of technique failure over the next 90 days. IFN-γ, interferon-γ; IL, interleukin; MMP, matrix metalloproteinase; sIL-6R, soluble IL-6 receptor; TGF-β, transforming growth factor-β; TNF-α, tumor necrosis factor-α; VEGF, vascular endothelial growth factor.

Taken together, our findings show that combinations of local biomarkers readily identify clinically meaningful subgroups of peritonitis patients, depending on the culture results and subsequent clinical outcomes (Figure 7). With microbiologically distinct infections also displaying immunologically distinct immune responses, most individual parameters constituting meaningful fingerprints were only associated with single patient groups. However, certain biomarkers featured more prominently in these mathematical models than others, suggesting that they are particularly important factors in acute peritonitis, such as IL-1β and IL-15, each of which contributed to 3 different biomarker signatures, and the total cell count that was included in 4 different signatures and is thus likely to be of the utmost relevance in the diagnosis of PD patients.

## Discussion

This study demonstrates that different groups of bacteria induce qualitatively distinct local immune responses in infected patients. Specific biomarker signatures were associated with acute infections by Gram-negative and Gram-positive organisms, respectively, and with culture-negative episodes of peritonitis of unclear etiology. Taking advantage of the unique access to local inflammatory responses that is possible in PD patients via the peritoneal catheter and drained dialysate, we were also able for the first time to characterize pathogen-specific local immune responses to defined genera of organisms such as streptococcal species and coagulase-negative staphylococci. These findings demonstrate the feasibility of developing rapid pathogen-specific diagnostics that exploit the exquisite responsiveness and specificity of the human immune system for different types of organisms. Such rapid methods might have greater utility than microbiological and molecular methods that aim at directly detecting such organisms but that are too slow, subject to confounding contaminants and often lack sufficient sensitivity to inform early patient management and target antibiotic therapy on the day of presentation with acute disease.^31^

Our study also demonstrates the power of using nonlinear approaches for mining complex biomedical datasets where conventional statistical methods fail to yield satisfactory results and where individual biomarkers on their own are unlikely to reach sufficient sensitivity and specificity to change clinical practice. Notably, the nature of the signatures identified in this study varied according to the mathematical model applied. By directly comparing 3 different approaches and assessing their performance when predicting microbiological and clinical endpoints in PD patients, we identified RFs as the most suitable models in this study and patient cohort, yielding superior performances and at fewer biomarkers than SVMs and ANNs, in accordance with investigations in other fields of research.^32^, ^33^, ^34^

Gram-negative bacteria, streptococci, and CNS are the major types of bacteria causing peritonitis in individuals receiving PD.^11^ Strikingly, clinical outcomes of infections by those organisms differ, implying differences in their pathogenicity, their susceptibility to antibiotics, and/or the pathophysiology of the host responses they trigger.^12^, ^15^ Our findings demonstrate that in addition to their microbiological differences, Gram-negative bacteria, streptococci, and CNS elicit fundamentally distinct immune responses, which is not only of relevance for the development of novel diagnostics but potentially also highlights key factors and cell types involved in sensing and fighting such infections. In this context, Vγ9/Vδ2 T cells and TNF-α appear to be particularly relevant in Gram-negative infections, IL-1β and MMPs in streptococcal infections, and IL-16 and sIL-6R in CNS infections. IL-15 featured in prediction models for streptococcal, nonstreptococcal, and CNS infection, indicating that this cytokine may play a role in all Gram-positive infections. In addition, the fact that IL-17A and interferon-γ featured prominently in nonstreptococcal Gram-positive infections (which included CNS), but not in CNS infections themselves, may argue for a particular role of both cytokines in response to Gram-positive bacteria other than streptococci and CNS (i.e., in infections by *Staphylococcus aureus* and/or coryneform bacteria). The organism-specific contributions of these soluble mediators during acute disease, the cell types that produce them, and the targets that respond to them can now be addressed in appropriate cellular assays, suitable animal models that mimic the situation in patients as closely as possible, and well-defined cohorts with bacterial peritonitis and other infections with access to the site of inflammation. This will ultimately further our understanding of antimicrobial immune responses and how to exploit such knowledge diagnostically and therapeutically. The roles of calprotectin, MMP-8, sIL-6R, and transforming growth factor-β as well as the balance between CD4^+^ and CD8^+^ T cells may deserve special attention with regard to their involvement in regulating pathologic processes in the peritoneal cavity, and their contribution to predicting clinical outcomes.

Taken together, we successfully applied different machine learning models to complex biomedical datasets and identified key pathways involved in pathogen-specific immune responses at the site of infection. It is apparent that the nature of the signatures identified depends on both biological and analytical parameters. However, our current findings demonstrate that such methodologies have immediate diagnostic and prognostic implications at the point of care, by informing patient management and the choice of treatment before traditional culture results become available. Being based on a relatively small population in a single hospital, the biomarkers identified in this study and the corresponding algorithms now await external validation in larger patient cohorts at multiple sites^35^ in order to demonstrate the applicability of the chosen approach to other centers where the spectrum of the infecting organisms and the previous infection history as well as patient demographics and health care settings may vary. Validated biomarker combinations can then be incorporated into appropriate diagnostic tests to be used in central laboratories or at the point of care and into new patient management and treatment guidelines based on such test results. The final choice of biomarkers to be taken forward will depend on the desired performance requirements, with soluble proteins being equally suitable for automated immunodiagnostic analyzers and bedside or home tests, whilst assessments of immune cell subsets such as Vγ9/Vδ2 T cells would require standardized flow cytometric protocols. In the meantime, our study reaffirms the importance of correctly interpreting simple parameters such as the total cell count (contributing to 4 different immune fingerprints) and the differential leukocyte count (reflected in the proportion of CD14^+^ cells among total cells), which already convey vital information about the nature of the causative pathogen.

## Materials and Methods

### Patient samples

This study was approved by the South East Wales Local Ethics Committee (04WSE04/27) and registered on the UK Clinical Research Network Study Portfolio under reference number #11838 “Patient Immune Responses to Infection in PD.” All individuals provided written informed consent. The local cohort comprised 83 adults PD patients admitted between 2008 and 2016 to the University Hospital of Wales, Cardiff, on day 1 of acute peritonitis. Twenty-four age- and sex-matched stable PD patients with no infection in the previous 3 months were included as controls. Subjects positive for HIV or hepatitis C virus were excluded.

Clinical diagnosis of acute peritonitis was based on the presence of abdominal pain and cloudy peritoneal effluent with >100 white blood cells per cubic millimeter. According to the microbiological analysis of the effluent from preinoculated blood culture bottles by the routine Microbiology Laboratory, Public Health Wales, peritonitis episodes were defined as culture-negative (*N* = 19, after incubation of up to 5 days) or as confirmed bacterial infections by Gram-positive (*N* = 47) and Gram-negative organisms (*N* = 17) (Table 1). Cases of fungal infection and mixed or unclear culture results were excluded from the study. Clinical outcomes were recorded by following patients for 90 days after presenting with peritonitis. Technique failure was defined as catheter removal, transfer to hemodialysis or death within 90 days, and occurred in 21.1% of culture-negative episodes, 23.4% of Gram-positive infections, and 47.1% of Gram-negative infections (Table 1). Samples from ≥8-hour dwells with volumes of 1 to 2.5 L were collected for biomarker measurements and processed as previously described.^6^, ^13^, ^14^, ^15^

### Cellular biomarkers

Cells from cloudy peritoneal effluents were acquired on an 8-color FACSCanto II (BD Biosciences, San Diego, CA) and analyzed with FlowJo 10.1 (TreeStar, Ashland, OR), using monoclonal antibodies against CD3 (SK7), CD4 (RPA-T4), CD8 (RPA-T8), CD15 (HI98 or HIM1), and TCR-Vδ2 from BD Biosciences; anti-TCR-Vγ9 (Immu360) from Beckman Coulter (Brea, CA); and anti-CD14 (61D3) from eBioscience (San Diego, CA); together with appropriate isotype controls. Leukocyte populations were gated based on their appearance in side scatter and forward scatter area/height and exclusion of live/dead staining (Fixable Aqua; Invitrogen, Carlsbad, CA). Biomarkers determined were the total cell counts; the percentages of CD3^+^ T cells, CD14^+^ monocytes/macrophages, and CD15^+^ neutrophils among total cells; the frequencies of CD4^+^, CD8^+^, Vγ9^+^, and Vδ2^+^ cells within the CD3^+^ T cell gate; and the ratios of CD4^+^ to CD8^+^ T cells.

### Soluble biomarkers

Cell-free peritoneal effluents were analyzed on a SECTOR Imager 6000 (Meso Scale Discovery, Rockville, MD) using the V-PLEX Human Cytokine 30-Plex Kit to measure levels of IL-1α, IL-1β, IL-2, IL-4, IL-5, IL-6, IL-7, IL-10, IL-12p40, IL-12p70, IL-13, IL-15, IL-16, IL-17A, interferon-γ, TNF-α, TNF-β, granulocyte macrophage colony-stimulating factor, and VEGF as well as the chemokines CCL2, CCL3, CCL4, CCL11, CCL13, CCL17, CCL22, CCL26, CXCL8, and CXCL10; ultrasensitive single-plex assays for sIL-6R and IL-18 (Meso Scale Discovery); and a customer-made single-plex assay for IL-22 using capture (MAB7822) and biotinylated detection antibodies (BAM7821) and recombinant human IL-22 from R&D Systems. Conventional enzyme-linked immunosorbent assay kits were used to measure transforming growth factor-β, total MMP-8, total MMP-9, and surfactant protein D (R&D Systems); calprotectin (Hycult Biotech, Inc., Plymouth Meeting, PA); and CCL2 (BD Biosciences). Human neutrophil elastase was measured using a B.I.T.S. enzyme-linked immunosorbent assay kit (Mologic, Bedford, UK). Active human neutrophil elastase was measured using the fluorogenic substrate MeOSuc-Ala-Ala-Pro-Val-AMC (Bachem, Bubendorf, Switzerland); active MMP was measured using the fluorogenic substrate Mca-Lys-Pro-Leu-Gly-Leu-Dpa-Ala-Arg-NH_2_ (Enzo Life Sciences, Farmingdale, NY) and by zymography using precast Novex gelatin zymogram gels (Invitrogen) scanned on a Bio-Rad GS800 densitometer (Bio-Rad Laboratories, Berkeley, CA) and analyzed using ImageJ software. Total protein was measured using the Pierce BCA Protein Assay Kit (Thermo Fisher Scientific, Waltham, MA). Measurements that were below or above the detection limit were replaced by the lowest and highest detectable values for each biomarker, respectively.

### Data preprocessing

All analyses were performed using R software version 3.2.5 (R Foundation, Vienna, Austria). Before applying machine learning models, missing data imputation was applied to fit gaps due to missing or failed measurements by adopting Multivariate Imputation by Chained Equations,^36^ which impute an incomplete feature by generating synthetic values taking into account their relationship with other biomarkers, using RF models (Supplementary Table S9). Data were then standardized to a mean of 0 and a variance of 1 to reduce the effect of large feature range variation. After preprocessing, the samples in the minority groups were unsampled so that minority and majority groups had equal frequencies.

### Feature elimination

The caret package in R^37^ was adopted for the implementation of recursive feature elimination methods using 3 different machine learning models: SVMs with radial basis function kernel in the kernlab R package,^38^ RFs with Breiman’s algorithm in the randomForest R package,^25^ and single-hidden-layer ANNs in the nnet R package.^39^ To reduce variability, resampling methods were applied in the outer layer of the iteration, and cross-validation was used in the model fitting and parameter tuning to avoid overfitting. During parameter tuning, the search regions for hyperparameters in different classification models were set as follows: the penalty factor *C* that controls the tradeoff between learning errors and the complexity term and the radial basis function kernel parameter σ both ranged from 2^−5^ to 2^5^ with steps of 2; the number of trees in RFs were selected from [100, 300, 500, 1000, 3000], and the size of ANNs ranged from 2 to 2^10^ hidden nodes with steps of 2 and a weight decay from 10^−4^ to 10^−10^ with steps of 10. The number of repeats for resampling was set as 5 and for cross-validation and model selection as 10.

### Basic statistical analyses

Correlations between all 49 biomarkers were determined using the corrplot R package,^40^ based on correlation calculations using the Hmisc R package.^41^ Means, SEs, skewness, and kurtosis were determined with plotrix R^42^ and e1071 R packages.^43^ Mann-Whitney tests (2-sample Wilcoxon tests) were applied to assess the relationship between two patient groups. Heat maps were visualized using the gplots R package.^44^

## Disclosure

C-YL, NT, and ME are inventors on patent applications filed by University College Cardiff Consultants Ltd. (Cardiff University) in Europe and the United States on the identification of bacterial infections in peritoneal dialysis patients. PD is the cofounder and chief scientific officer of Mologic Ltd.; PD and GP are shareholders/option holders in Mologic. All the other authors declared no competing interests.

## Supplementary Material

Figure S1 Local immune fingerprints in Gram-positive infections. (**A**) Performance of Random Forest (RF), Support Vector Machine (SVM), and artificial neural network (ANN)–based feature elimination models for the prediction of Gram-positive infections (*N* = 47) against all other episodes of peritonitis (*N* = 36), shown as area under the curve (AUC) depending on the number of biomarkers. Red symbols depict the maximum AUC for each model. (**B**) Kurtosis and skewness of the top 5 biomarkers selected by RF-based feature elimination. (**C**) Receiver operating characteristic analysis showing specificity and sensitivity of the top 5 biomarkers. (**D**) Tukey plots of the top 5 biomarkers in patients with confirmed Gram-positive infections and with all other episodes of peritonitis, as assessed by Mann-Whitney tests (**P* < 0.05; ***P* < 0.01; ****P* < 0.001). (**E**) Heat map showing the top 5 biomarkers across all patients presenting with acute peritonitis. IFN-γ, interferon-γ; IL, interleukin.

Figure S2 Local immune fingerprints in nonstreptococcal Gram-positive infections. (**A**) Performance of Random Forest (RF), Support Vector Machine (SVM), and artificial neural network (ANN)–based feature elimination models, for the prediction of infections caused by nonstreptococcal Gram-positive species (*Staphylococcus aureus*, coagulase-negative *Staphylococcus* spp., *Corynebacterium* spp.; *N* = 31) against all other episodes of peritonitis (*N* = 62), shown as area under the curve (AUC) depending on the number of biomarkers. One episode of peritonitis classified as nonstreptococcal (Non-strep) infection was a coinfection caused by *Corynebacterium* sp. and coagulase-negative *Staphylococcus* sp. Red symbols depict the maximum AUC for each model. (**B**) Kurtosis and skewness of the top 5 biomarkers selected by RF-based feature elimination. (**C**) Receiver operating characteristic analysis showing specificity and sensitivity of the top 5 biomarkers. (**D**) Tukey plots of the top 5 biomarkers in patients with confirmed nonstreptococcal Gram-positive infections and with all other episodes of peritonitis, as assessed by Mann-Whitney tests (**P* < 0.05; ***P* < 0.01; ****P* < 0.001). (**E**) Heat map showing the top 5 biomarkers across all patients presenting with acute peritonitis.

Table S1 Local biomarkers in stable PD patients and in patients presenting with acute peritonitis.

Table S2A Local biomarkers in patients presenting with acute peritonitis caused by Gram-negative organisms or with other episodes.

Table S2B Performance of local biomarkers in predicting Gram-negative infections in PD patients against all other episodes of peritonitis.

Table S3A Local biomarkers in patients presenting with culture-negative peritonitis or with other episodes.

Table S3B Performance of local biomarkers in predicting culture-negative episodes in PD patients against all other microbiologically confirmed infections.

Table S4A Local biomarkers in patients presenting with acute peritonitis caused by Gram-positive organisms or with other episodes.

Table S4B Performance of local biomarkers in predicting Gram-positive infections in PD patients against all other episodes of peritonitis.

Table S5A Local biomarkers in patients presenting with acute peritonitis caused by streptococcal species (*Streptococcus* spp. and *Enterococcus* spp.) or with other episodes.

Table S5B Performance of local biomarkers in predicting infections caused by streptococcal species (*Streptococcus* spp. and *Enterococcus* spp.) against all other episodes of peritonitis.

Table S6A Local biomarkers in patients presenting with acute peritonitis caused by nonstreptococcal Gram-positive species (*Staphylococcus aureus*, coagulase-negative *Staphylococcus* spp., *Corynebacterium* spp.) or with other episodes.

Table S6B Performance of local biomarkers in predicting infections caused by nonstreptococcal Gram-positive species (*Staphylococcus aureus*, coagulase-negative *Staphylococcus spp.*, *Corynebacterium spp.*) against all other episodes of peritonitis.

Table S7A Local biomarkers in patients presenting with acute peritonitis caused by coagulase-negative staphylococci or with other episodes.

Table S7B Performance of local biomarkers in predicting infections caused by coagulase-negative staphylococci against all other episodes of peritonitis.

Table S8A Local biomarkers in patients technique failure over the next 90 days or with other episodes.

Table S8B Performance of local biomarkers in predicting technique failure over the next 90 days against all other episodes of peritonitis.

Table S9 Proportion of missing values.
