# Supplementary material for: Machine-learning algorithms define pathogen-specific local immune fingerprints in peritoneal dialysis patients with bacterial infections
Source: Kidney Int. 2017 Jul;92(1):179–91. doi: 10.1016/j.kint.2017.01.017 (PMC5484022; doi:10.1016/j.kint.2017.01.017)
Supplement: Figure S1 — Local immune fingerprints in Gram-positive infections. (A) Performance of Random Forest (RF), Support Vector Machine (SVM), and artificial neural network (ANN)–based feature elimination models for the prediction of Gram-positive infections (N = 47) against all other episodes of peritonitis (N = 36), shown as area under the curve (AUC) depending on the number of biomarkers. Red symbols depict the maximum AUC for each model. (B) Kurtosis and skewness of the top 5 biomarkers selected by RF-based feature elimination. (C) Receiver operating characteristic analysis showing specificity and sensitivity of the top 5 biomarkers. (D) Tukey plots of the top 5 biomarkers in patients with confirmed Gram-positive infections and with all other episodes of peritonitis, as assessed by Mann-Whitney tests (*P < 0.05; **P < 0.01; ***P < 0.001). (E) Heat map showing the top 5 biomarkers across all patients presenting with acute peritonitis. IFN-γ, interferon-γ; IL, interleukin. [file mmc1.docx]

**Supplementary Figure S1. Local immune fingerprints in Gram-positive infections.** (***A***) Performance of RF, SVM and ANN based feature elimination models, for the prediction of Gram-positive infections (*n*=47) against all other episodes of peritonitis (*n*=36), shown as AUC depending on the number of biomarkers. Red symbols depict the maximum AUC for each model. (***B***) Kurtosis and skewness of the top 5 biomarkers selected by RF-based feature elimination. (***C***) ROC analysis showing specificity and sensitivity of the top 5 biomarkers. (***D***) Tukey plots of the top 5 biomarkers in patients with confirmed Gram-positive infections and with all other episodes of peritonitis, as assessed by Mann-Whitney tests (*, *p*<0.05; **, *p*<0.01; ***, *p*<0.001). (***E***) Heatmap showing the top 5 biomarkers across all patients presenting with acute peritonitis.
