# Supplementary material for: Machine-learning algorithms define pathogen-specific local immune fingerprints in peritoneal dialysis patients with bacterial infections
Source: Kidney Int. 2017 Jul;92(1):179–91. doi: 10.1016/j.kint.2017.01.017 (PMC5484022; doi:10.1016/j.kint.2017.01.017)
Supplement: Table S1 — Local biomarkers in stable PD patients and in patients presenting with acute peritonitis. [file mmc3.docx]

Supplementary Table S1. Local biomarkers in stable PD patients and in patients presenting with acute peritonitis.

| Biomarker | **Stable** | | **Peritonitis** | | *p* |
| --- | --- | --- | --- | --- | --- |
|  | Mean | *SEM* | Mean | *SEM* |  |
| IL-1α (pg/ml) | 22.47 | *0.00* | 27.92 | *3.38* |  |
| IL-1β (pg/ml) | 1.04 | *0.24* | 34.60 | *8.18* | *** |
| IL-2 (pg/ml) | 1.30 | *0.16* | 10.46 | *2.30* | *** |
| IL-4 (pg/ml) | 0.35 | *0.06* | 3.56 | *0.39* | *** |
| IL-5 (pg/ml) | 3.60 | *0.39* | 2.27 | *0.39* | *** |
| IL-6 (pg/ml) | 172.99 | *42.37* | 766.19 | *21.28* | *** |
| IL-7 (pg/ml) | 9.77 | *0.84* | 3.90 | *0.43* | *** |
| IL-10 (pg/ml) | 1.97 | *0.27* | 46.86 | *7.89* | *** |
| IL-12p40 (pg/ml) | 98.45 | *11.62* | 200.90 | *45.39* |  |
| IL-12p70 (pg/ml) | 1.22 | *0.10* | 7.01 | *0.80* | *** |
| IL-13 (pg/ml) | 8.54 | *0.98* | 20.80 | *2.16* | *** |
| IL-15 (pg/ml) | 6.65 | *0.49* | 5.84 | *0.74* | ** |
| IL-16 (pg/ml) | 118.62 | *11.36* | 482.04 | *64.47* | *** |
| IL-17A (pg/ml) | 4.27 | *0.62* | 76.60 | *22.67* | *** |
| IL-18 (pg/ml) | 89.42 | *15.29* | 91.62 | *19.56* | ** |
| IL-22 (pg/ml) | 25.99 | *0.12* | 30.07 | *1.41* |  |
| sIL-6R (pg/ml) | 793.97 | *66.76* | 1548.54 | *73.19* | *** |
| IFN-γ (pg/ml) | 10.93 | *1.83* | 168.06 | *36.93* | *** |
| TNF-α (pg/ml) | 3.91 | *0.89* | 89.42 | *14.09* | *** |
| TNF-β (pg/ml) | 0.42 | *0.02* | 0.97 | *0.34* |  |
| GM-CSF (pg/ml) | 2.03 | *0.34* | 1.95 | *0.29* | * |
| TGF-β (pg/ml) | 63.48 | *10.86* | 241.82 | *17.88* | *** |
| VEGF (pg/ml) | 142.91 | *15.11* | 163.12 | *25.98* | * |
| CCL2 (pg/ml) | 551.40 | *6.10* | 482.78 | *15.70* |  |
| CCL3 (pg/ml) | 46.90 | *5.62* | 309.96 | *44.28* | *** |
| CCL4 (pg/ml) | 83.34 | *14.92* | 675.52 | *54.70* | *** |
| CCL11 (pg/ml) | 572.98 | *76.20* | 1076.20 | *59.79* | *** |
| CCL13 (pg/ml) | 76.30 | *10.27* | 39.44 | *5.28* | *** |
| CCL17 (pg/ml) | 61.47 | *5.20* | 118.18 | *22.24* |  |
| CCL22 (pg/ml) | 578.21 | *47.81* | 491.38 | *47.75* | ** |
| CCL26 (pg/ml) | 206.75 | *184.31* | 69.80 | *6.74* | *** |
| CXCL8 (pg/ml) | 183.86 | *95.61* | 4083.34 | *1268.60* | ** |
| CXCL10 (pg/ml) | 557.10 | *54.85* | 1945.29 | *116.80* | *** |
| MMP-8 total (ng/ml) | 0.04 | *0.01* | 23.88 | *1.67* | *** |
| MMP substrate (ng/ml) | 11.10 | *1.22* | 17.39 | *1.44* |  |
| Human neutrophil elastase (ng/ml) | 1.49 | *0.56* | 137.99 | *10.21* | *** |
| HNE substrate (ng/ml) | 6.00 | *2.77* | 12.64 | *2.06* |  |
| Zymography (arbitrary units) | 0.39 | *0.05* | 1.89 | *0.11* | *** |
| Calprotectin (ng/ml) | 3.43 | *0.89* | 81.27 | *1.94* | *** |
| Surfactant protein D (SPD) | 1.23 | *0.21* | 1.59 | *0.11* |  |
| Total cell count (× 10^9^ cells) | 0.04 | *0.03* | 7.73 | *1.33* | *** |

Differences between stable patients and patients with acute peritonitis were considered statistically significant as indicated:
* *p*<0.05, ** *p*<0.01, *** *p*<0.001, based on two-tailed Mann-Whitney tests.

Differences in the cellular composition between stable PD patients and patients presenting with peritonitis were already published earlier (Lin *et al.*, *J Am Soc Nephrol* **24**:2002-2009).
