# Supplementary material for: Machine-learning algorithms define pathogen-specific local immune fingerprints in peritoneal dialysis patients with bacterial infections
Source: Kidney Int. 2017 Jul;92(1):179–91. doi: 10.1016/j.kint.2017.01.017 (PMC5484022; doi:10.1016/j.kint.2017.01.017)
Supplement: Table S2A — Local biomarkers in patients presenting with acute peritonitis caused by Gram-negative organisms or with other episodes. [file mmc4.docx]

Supplementary Table S2A. Local biomarkers in patients presenting with acute peritonitis caused by Gram-negative organisms or with other episodes.

| Biomarker | **Gram-negative infections** | | **Other episodes** | | *p* |
| --- | --- | --- | --- | --- | --- |
|  | Mean | *SEM* | Mean | *SEM* |  |
| IL-1α (pg/ml) | 31.33 | *6.29* | 27.05 | *3.94* | * |
| IL-1β (pg/ml) | 53.74 | *14.11* | 29.67 | *9.58* | ** |
| IL-2 (pg/ml) | 8.45 | *1.95* | 10.97 | *2.85* |  |
| IL-4 (pg/ml) | 5.35 | *1.07* | 3.09 | *0.39* | * |
| IL-5 (pg/ml) | 2.25 | *0.66* | 2.27 | *0.46* |  |
| IL-6 (pg/ml) | 821.73 | *11.27* | 751.88 | *26.36* |  |
| IL-7 (pg/ml) | 4.22 | *1.09* | 3.81 | *0.47* |  |
| IL-10 (pg/ml) | 91.54 | *22.77* | 35.3 | *7.48* | ** |
| IL-12p40 (pg/ml) | 108.49 | *24.88* | 224.71 | *56.46* |  |
| IL-12p70 (pg/ml) | 9.69 | *2.09* | 6.33 | *0.84* | * |
| IL-13 (pg/ml) | 29.25 | *7.68* | 18.62 | *1.81* | * |
| IL-15 (pg/ml) | 6.50 | *1.93* | 5.67 | *0.79* |  |
| IL-16 (pg/ml) | 541.76 | *152.03* | 466.66 | *71.49* |  |
| IL-17A (pg/ml) | 37.95 | *16.03* | 86.55 | *28.14* |  |
| IL-18 (pg/ml) | 80.58 | *23.31* | 94.38 | *23.80* |  |
| IL-22 (pg/ml) | 29.08 | *3.13* | 30.33 | *1.59* |  |
| sIL-6R (pg/ml) | 1840.15 | *149.31* | 1473.43 | *81.62* | 0.051 |
| IFN-γ (pg/ml) | 52.75 | *12.37* | 197.76 | *45.69* |  |
| TNF-α (pg/ml) | 172.37 | *35.54* | 68.06 | *14.17* | ** |
| TNF-β (pg/ml) | 0.46 | *0.08* | 1.10 | *0.42* |  |
| GM-CSF (pg/ml) | 1.97 | *0.50* | 1.94 | *0.34* |  |
| TGF-β (pg/ml) | 215.50 | *33.32* | 248.60 | *20.81* |  |
| VEGF (pg/ml) | 221.88 | *74.13* | 147.99 | *26.55* |  |
| CCL2 (pg/ml) | 517.91 | *27.83* | 473.73 | *18.32* |  |
| CCL3 (pg/ml) | 616.44 | *117.56* | 231.02 | *42.02* | ** |
| CCL4 (pg/ml) | 964.73 | *84.99* | 601.02 | *62.23* | ** |
| CCL11 (pg/ml) | 1198.90 | *115.74* | 1044.60 | *68.90* |  |
| CCL13 (pg/ml) | 60.25 | *19.82* | 34.08 | *4.15* |  |
| CCL17 (pg/ml) | 108.79 | *27.20* | 120.60 | *27.15* |  |
| CCL22 (pg/ml) | 536.66 | *131.34* | 479.72 | *50.13* |  |
| CCL26 (pg/ml) | 98.65 | *15.70* | 62.37 | *7.23* | * |
| CXCL8 (pg/ml) | 9988.27 | *5380.25* | 2562.37 | *737.63* |  |
| CXCL10 (pg/ml) | 1807.11 | *275.16* | 1980.89 | *129.42* |  |
| MMP-8 total (ng/ml) | 33.21 | *4.05* | 21.65 | *1.73* | ** |
| MMP substrate (ng/ml) | 20.52 | *3.73* | 16.65 | *1.55* |  |
| Human neutrophil elastase (ng/ml) | 130.71 | *19.14* | 139.72 | *11.84* |  |
| HNE substrate (ng/ml) | 9.87 | *3.74* | 13.30 | *2.40* |  |
| Zymography (arbitrary units) | 1.90 | *0.32* | 1.89 | *0.11* |  |
| Calprotectin (ng/ml) | 83.93 | *3.47* | 80.63 | *2.25* |  |
| Surfactant protein D (SPD) | 1.48 | *0.19* | 1.61 | *0.13* |  |
| Total cell count (× 10^9^ cells) | 9.69 | *2.04* | 7.23 | *1.59* | * |
| CD3^+^ (% of total) | 0.37 | *0.11* | 1.39 | *0.31* | * |
| CD14^+^ (% of total) | 6.97 | *1.37* | 13.24 | *1.57* | * |
| CD15^+^ (% of total) | 85.52 | *2.03* | 77.75 | *2.03* | 0.050 |
| CD4:CD8 ratio | 1.23 | *0.24* | 1.62 | *0.16* |  |
| CD4^+^ (% of T cells) | 43.22 | *3.36* | 49.86 | *2.00* |  |
| CD8^+^ (% of T cells) | 42.61 | *3.50* | 38.44 | *1.71* |  |
| Vγ9^+^ (% of T cells) | 5.47 | *1.15* | 2.40 | *0.28* | ** |
| Vδ2^+^ (% of T cells) | 6.43 | *1.29* | 2.73 | *0.43* | ** |

Differences between Gram-negative and other episodes were considered statistically significant as indicated:
* *p*<0.05, ** *p*<0.01, *** *p*<0.001, based on two-tailed Mann-Whitney tests.
