# Supplementary material for: Machine-learning algorithms define pathogen-specific local immune fingerprints in peritoneal dialysis patients with bacterial infections
Source: Kidney Int. 2017 Jul;92(1):179–91. doi: 10.1016/j.kint.2017.01.017 (PMC5484022; doi:10.1016/j.kint.2017.01.017)
Supplement: Table S2B — Performance of local biomarkers in predicting Gram-negative infections in PD patients against all other episodes of peritonitis. [file mmc5.docx]

Supplementary Table S2B. Performance of local biomarkers in predicting Gram-negative infections in PD patients against all other episodes of peritonitis.

| **Model** | **Size** | **Biomarker(s)** | **AUC** | **Sensitivity** | **Specificity** | |
| --- | --- | --- | --- | --- | --- | --- |
| ANN | 5 | Vδ2^+^, CD14^+^, CCL2, Vγ9^+^, IL-10 | 0.891 ± *0.054* | 0.97 ± *0.04* | 0.77 ± *0.08* |  |
|  | 10 | + SPD, CCL3, MMP substrate, VEGF, IL-5 | 0.921 ± *0.092* | 0.99 ± *0.03* | 0.77 ± *0.18* |  |
| SVM | 5 | Vδ2^+^, TNF-α, CCL3, Vγ9^+^, MMP-8 | 0.989 ± *0.171* | 0.96 ± *0.04* | 0.99 ± *0.03* |  |
|  | 10 | + IL-1β, CCL4, IL-10, CD14^+^, IL-4 | 0.995 ± *0.009* | 0.74 ± *0.43* | 0.80 ± *0.45* |  |
| RF | 5 | Vδ2^+^, IL-12p40, Vγ9^+^, VEGF, TNF-α | 0.987 ± *0.021* | 0.99 ± *0.03* | 0.87 ± *0.12* |  |
|  | 10 | + MMP-8, CCL4, CD14^+^, CCL3, zymography | 0.990 ± *0.018* | 0.99 ± *0.03* | 0.88 ± *0.07* |  |
| ROC | 1 | Vδ2^+^, cut-off: 2.1 % of T cells | 0.78 *(0.64–0.91)* | 0.86 | 0.60 |  |
|  | 1 | IL-12p40, cut-off: 55.7 pg/ml | 0.58 *(0.43–0.74)* | 0.53 | 0.71 |  |
|  | 1 | Vγ9^+^, cut-off: 3.2 % of T cells | 0.76 *(0.62–0.90)* | 0.67 | 0.78 |  |
|  | 1 | VEGF, cut-off: 279.9 pg/ml | 0.50 *(0.31–0.70)* | 0.29 | 0.89 |  |
|  | 1 | TNF-α, cut-off: 22.8 pg/ml | 0.75 *(0.62–0.88)* | 0.82 | 0.65 |  |

Shown are the biomarker combinations as selected by recursive feature elimination using RF, SVM and ANN models, listed in the order of the importance in the different models. The top 5 biomarkers from the RF model were also evaluated individually in conventional ROC analyses. AUC, specificity and sensitivity for machine learning model are shown as average and *SEM* values of the validation dataset after five rounds of re-sampling. Values for individual markers are shown as AUC with lower and higher confidence boundaries. Cut-off values were determined from the highest sum of sensitivity and specificity.
