# Supplementary material for: Machine-learning algorithms define pathogen-specific local immune fingerprints in peritoneal dialysis patients with bacterial infections
Source: Kidney Int. 2017 Jul;92(1):179–91. doi: 10.1016/j.kint.2017.01.017 (PMC5484022; doi:10.1016/j.kint.2017.01.017)
Supplement: Table S3A — Local biomarkers in patients presenting with culture-negative peritonitis or with other episodes. [file mmc6.docx]

Supplementary Table S3A: Local biomarkers in patients presenting with culture-negative peritonitis or with other episodes.

| Biomarker | **No growth** | | **Other episodes** | | *p* |
| --- | --- | --- | --- | --- | --- |
|  | Mean | *SEM* | Mean | *SEM* |  |
| IL-1α (pg/ml) | 22.47 | *0* | 29.54 | *4.37* |  |
| IL-1β (pg/ml) | 4.87 | *3.22* | 43.42 | *10.33* | *** |
| IL-2 (pg/ml) | 4.33 | *0.97* | 12.28 | *2.94* | ** |
| IL-4 (pg/ml) | 1.5 | *0.42* | 4.17 | *0.46* | *** |
| IL-5 (pg/ml) | 4.18 | *1.48* | 1.70 | *0.22* |  |
| IL-6 (pg/ml) | 606.24 | *72.15* | 813.67 | *12.89* | *** |
| IL-7 (pg/ml) | 5.2 | *1.39* | 3.51 | *0.38* |  |
| IL-10 (pg/ml) | 12.66 | *4.9* | 57.01 | *9.79* | *** |
| IL-12p40 (pg/ml) | 72.47 | *15.51* | 239.03 | *57.94* | ** |
| IL-12p70 (pg/ml) | 3.63 | *0.96* | 8.02 | *0.96* | ** |
| IL-13 (pg/ml) | 14.17 | *2.33* | 22.77 | *2.67* | * |
| IL-15 (pg/ml) | 5.09 | *1.27* | 6.07 | *0.89* |  |
| IL-16 (pg/ml) | 319.46 | *106.3* | 530.31 | *76.78* | * |
| IL-17A (pg/ml) | 10.71 | *5.6* | 96.16 | *28.96* | *** |
| IL-18 (pg/ml) | 77.93 | *26.5* | 95.88 | *24.36* |  |
| IL-22 (pg/ml) | 26.5 | *0.63* | 31.22 | *1.83* |  |
| sIL-6R (pg/ml) | 1315.71 | *139.92* | 1617.66 | *83.97* |  |
| IFN-γ (pg/ml) | 62.42 | *42.09* | 199.42 | *45.65* | *** |
| TNF-α (pg/ml) | 13.96 | *4.01* | 111.82 | *17.30* | *** |
| TNF-β (pg/ml) | 0.48 | *0.06* | 1.12 | *0.44* |  |
| GM-CSF (pg/ml) | 1.49 | *0.34* | 2.09 | *0.36* |  |
| TGF-β (pg/ml) | 276.26 | *45.41* | 231.60 | *18.90* |  |
| VEGF (pg/ml) | 90.31 | *39.74* | 184.74 | *31.19* | * |
| CCL2 (pg/ml) | 360.08 | *42.28* | 519.21 | *13.13* | *** |
| CCL3 (pg/ml) | 111.04 | *46.55* | 369.02 | *53.70* | *** |
| CCL4 (pg/ml) | 286.04 | *96.58* | 791.14 | *57.73* | *** |
| CCL11 (pg/ml) | 697.62 | *118.08* | 1188.59 | *63.06* | *** |
| CCL13 (pg/ml) | 31.77 | *9.47* | 41.72 | *6.26* |  |
| CCL17 (pg/ml) | 167.37 | *89.03* | 103.58 | *12.15* | * |
| CCL22 (pg/ml) | 413.99 | *110.34* | 514.36 | *52.72* | * |
| CCL26 (pg/ml) | 36.44 | *5* | 79.71 | *8.23* | *** |
| CXCL8 (pg/ml) | 639.78 | *252.54* | 5105.64 | *1624.41* | * |
| CXCL10 (pg/ml) | 1453.2 | *250.9* | 2091.39 | *127.32* | ** |
| MMP-8 total (ng/ml) | 12.08 | *2.53* | 27.41 | *1.81* | *** |
| MMP substrate (ng/ml) | 9.67 | *1.59* | 19.71 | *1.70* | *** |
| Human neutrophil elastase (ng/ml) | 88.24 | *18.84* | 152.91 | *11.39* | ** |
| HNE substrate (ng/ml) | 12.33 | *6.01* | 12.73 | *2.03* |  |
| Zymography (arbitrary units) | 1.47 | *0.17* | 2.02 | *0.13* | * |
| Calprotectin (ng/ml) | 77.4 | *4.96* | 82.43 | *2.03* |  |
| Surfactant protein D (SPD) | 1.37 | *0.17* | 1.65 | *0.14* |  |
| Total cell count (× 10^9^ cells) | 1.13 | *0.23* | 9.67 | *1.64* | *** |
| CD3^+^ (% of total) | 1.5 | *0.57* | 1.09 | *0.28* | 0.064 |
| CD14^+^ (% of total) | 22.41 | *4.24* | 9.02 | *0.87* | *** |
| CD15^+^ (% of total) | 67.45 | *4.4* | 82.69 | *1.54* | *** |
| CD4:CD8 ratio | 1.5 | *0.2* | 1.55 | *0.17* |  |
| CD4^+^ (% of T cells) | 49.96 | *4.38* | 0.20 | *1.90* |  |
| CD8^+^ (% of T cells) | 37.31 | *2.23* | 39.84 | *1.87* |  |
| Vγ9^+^ (% of T cells) | 2.91 | *0.66* | 3.06 | *0.41* |  |
| Vδ2^+^ (% of T cells) | 2.4 | *0.62* | 3.78 | *0.56* |  |

Differences between the two patient groups were considered statistically significant as indicated:
* *p*<0.05, ** *p*<0.01, *** *p*<0.001, based on two-tailed Mann-Whitney tests.
