# Supplementary material for: Machine-learning algorithms define pathogen-specific local immune fingerprints in peritoneal dialysis patients with bacterial infections
Source: Kidney Int. 2017 Jul;92(1):179–91. doi: 10.1016/j.kint.2017.01.017 (PMC5484022; doi:10.1016/j.kint.2017.01.017)
Supplement: Table S3B — Performance of local biomarkers in predicting culture-negative episodes in PD patients against all other microbiologically confirmed infections. [file mmc7.docx]

Supplementary Table S3B. Performance of local biomarkers in predicting culture-negative episodes in PD patients against all other microbiologically confirmed infections.

| **Model** | **Size** | **Biomarker(s)** | **AUC** | **Sensitivity** | **Specificity** |
| --- | --- | --- | --- | --- | --- |
| ANN | 5 | CD14^+^, IL-6, Vδ2^+^, CCL13, cell count | 0.920 ± *0.060* | 0.92 ± *0.12* | 0.86 ± *0.06* |
|  | 10 | + CD3^+^, IL-7, TNF-α, HNE, CCL2 | 0.941 ± *0.043* | 0.92 ± *0.08* | 0.81 ± *0.10* |
| SVM | 5 | IL-1β, cell count, MMP-8, CD14^+^, IL-4 | 0.893 ± *0.073* | 0.75 ± *0.43* | 0.69 ± *0.43* |
|  | 10 | + IL-17A, CCL4, IL-10, IFN-γ, TNF-α | 0.957 ± *0.066* | 0.78 ± *0.44* | 0.80 ± *0.45* |
| RF | 5 | Cell count, IL-1β, MMP-8, CD14^+^, CCL4 | 0.984 ± *0.021* | 0.94 ± *0.09* | 0.81 ± *0.16* |
|  | 10 | + CD15^+^, IL-4, IFN-γ, MMP substrate, CCL17 | 0.963 ± *0.048* | 0.94 ± *0.09* | 0.83 ± *0.21* |
| ROC | 1 | Cell count, cut-off: 2.7 × 10^9^ cells | 0.88 *(0.80–0.95)* | 0.94 | 0.72 |
|  | 1 | IL-1β, cut-off: 1.2 pg/ml | 0.86 *(0.76–0.96)* | 0.68 | 0.94 |
|  | 1 | MMP-8, cut-off: 10.9 ng/ml | 0.81 *(0.71–0.92)* | 0.67 | 0.85 |
|  | 1 | CD14^+^, cut-off: 19.3 % of total cells | 0.80 *(0.67–0.93)* | 0.56 | 0.93 |
|  | 1 | CCL4, cut-off: 145.2 pg/ml | 0.80 *(0.68–0.93)* | 0.68 | 0.84 |

Shown are the biomarker combinations as selected by recursive feature elimination using RF, SVM and ANN models, listed in the order of the importance in the different models. The top 5 biomarkers from the RF model were also evaluated individually in conventional ROC analyses. AUC, specificity and sensitivity for machine learning model are shown as average and *SEM* values of the validation dataset after five rounds of re-sampling. Values for individual markers are shown as AUC with lower and higher confidence boundaries. Cut-off values were determined from the highest sum of sensitivity and specificity.
