# Supplementary material for: Machine-learning algorithms define pathogen-specific local immune fingerprints in peritoneal dialysis patients with bacterial infections
Source: Kidney Int. 2017 Jul;92(1):179–91. doi: 10.1016/j.kint.2017.01.017 (PMC5484022; doi:10.1016/j.kint.2017.01.017)
Supplement: Table S4A — Local biomarkers in patients presenting with acute peritonitis caused by Gram-positive organisms or with other episodes. [file mmc8.docx]

Supplementary Table S4A. Local biomarkers in patients presenting with acute peritonitis caused by Gram-positive organisms or with other episodes.

| Biomarker | **Gram-positive infections** | | **Other episodes** | | *p* |
| --- | --- | --- | --- | --- | --- |
|  | Mean | *SEM* | Mean | *SEM* |  |
| IL-1α (pg/ml) | 28.9 | *5.53* | 26.65 | *3.02* |  |
| IL-1β (pg/ml) | 39.69 | *13.14* | 27.95 | *7.92* | 0.075 |
| IL-2 (pg/ml) | 13.66 | *3.93* | 6.27 | *1.10* |  |
| IL-4 (pg/ml) | 3.74 | *0.49* | 3.32 | *0.63* |  |
| IL-5 (pg/ml) | 1.5 | *0.18* | 3.27 | *0.85* |  |
| IL-6 (pg/ml) | 810.76 | *17.13* | 708.00 | *42.08* | * |
| IL-7 (pg/ml) | 3.25 | *0.33* | 4.74 | *0.89* |  |
| IL-10 (pg/ml) | 44.52 | *10.04* | 49.91 | *12.76* |  |
| IL-12p40 (pg/ml) | 286.25 | *77.46* | 89.48 | *14.43* | ** |
| IL-12p70 (pg/ml) | 7.41 | *1.08* | 6.49 | *1.20* |  |
| IL-13 (pg/ml) | 20.42 | *2.33* | 21.29 | *3.98* |  |
| IL-15 (pg/ml) | 5.91 | *1* | 5.76 | *1.12* |  |
| IL-16 (pg/ml) | 526.17 | *89.92* | 424.44 | *91.70* |  |
| IL-17A (pg/ml) | 117.21 | *38.67* | 23.57 | *8.32* | *** |
| IL-18 (pg/ml) | 101.32 | *32.07* | 79.14 | *17.65* |  |
| IL-22 (pg/ml) | 32.02 | *2.24* | 27.68 | *1.46* |  |
| sIL-6R (pg/ml) | 1537.19 | *99.08* | 1563.36 | *109.93* |  |
| IFN-γ (pg/ml) | 252.47 | *60.3* | 57.86 | *22.69* | ** |
| TNF-α (pg/ml) | 89.93 | *18.97* | 88.77 | *21.35* |  |
| TNF-β (pg/ml) | 1.36 | *0.59* | 0.47 | *0.05* |  |
| GM-CSF (pg/ml) | 2.13 | *0.46* | 1.72 | *0.30* |  |
| TGF-β (pg/ml) | 237.43 | *22.89* | 247.57 | *28.73* |  |
| VEGF (pg/ml) | 171.31 | *33.28* | 152.44 | *41.69* | 0.075 |
| CCL2 (pg/ml) | 519.68 | *14.97* | 434.61 | *28.81* | * |
| CCL3 (pg/ml) | 279.52 | *54.63* | 349.70 | *73.42* |  |
| CCL4 (pg/ml) | 728.36 | *70.59* | 606.53 | *85.86* |  |
| CCL11 (pg/ml) | 1184.87 | *75.69* | 934.34 | *92.01* | * |
| CCL13 (pg/ml) | 35.02 | *4.46* | 45.22 | *10.72* |  |
| CCL17 (pg/ml) | 101.7 | *13.49* | 139.70 | *48.33* |  |
| CCL22 (pg/ml) | 506.29 | *54.85* | 471.92 | *84.47* |  |
| CCL26 (pg/ml) | 72.86 | *9.56* | 65.82 | *9.36* |  |
| CXCL8 (pg/ml) | 3339.59 | *1011.9* | 5054.34 | *2624.60* |  |
| CXCL10 (pg/ml) | 2194.21 | *140.85* | 1620.32 | *185.26* | ** |
| MMP-8 total (ng/ml) | 25.48 | *1.94* | 21.69 | *2.93* |  |
| MMP substrate (ng/ml) | 19.44 | *1.93* | 14.60 | *2.10* | * |
| Human neutrophil elastase (ng/ml) | 13.69 | *2.4* | 11.21 | *3.65* | * |
| HNE substrate (ng/ml) | 2.06 | *0.13* | 1.67 | *0.18* | * |
| Zymography (arbitrary units) | 160.31 | *13.71* | 107.54 | *13.77* |  |
| Calprotectin (ng/ml) | 81.93 | *2.47* | 80.37 | *3.14* |  |
| Surfactant protein D (SPD) | 1.71 | *0.17* | 1.42 | *0.13* |  |
| Total cell count (× 10^9^ cells) | 9.67 | *2.12* | 5.16 | *1.21* | * |
| CD3^+^ (% of total) | 1.35 | *0.37* | 0.95 | *0.31* |  |
| CD14^+^ (% of total) | 9.75 | *1.06* | 14.94 | *2.66* |  |
| CD15^+^ (% of total) | 81.68 | *1.95* | 76.19 | *2.94* |  |
| CD4:CD8 ratio | 1.66 | *0.21* | 1.37 | *0.16* |  |
| CD4^+^ (% of T cells) | 49.83 | *2.24* | 46.71 | *2.81* |  |
| CD8^+^ (% of T cells) | 38.87 | *2.21* | 39.87 | *2.07* |  |
| Vγ9^+^ (% of T cells) | 2.2 | *0.29* | 4.15 | *0.68* | * |
| Vδ2^+^ (% of T cells) | 2.86 | *0.54* | 4.35 | *0.79* |  |

Differences between the two patient groups were considered statistically significant as indicated:
* *p*<0.05, ** *p*<0.01, *** *p*<0.001, based on two-tailed Mann-Whitney tests.
