# Supplementary material for: Machine-learning algorithms define pathogen-specific local immune fingerprints in peritoneal dialysis patients with bacterial infections
Source: Kidney Int. 2017 Jul;92(1):179–91. doi: 10.1016/j.kint.2017.01.017 (PMC5484022; doi:10.1016/j.kint.2017.01.017)
Supplement: Table S4B — Performance of local biomarkers in predicting Gram-positive infections in PD patients against all other episodes of peritonitis. [file mmc9.docx]

Supplementary Table S4B. Performance of local biomarkers in predicting Gram-positive infections in PD patients against all other episodes of peritonitis.

| **Model** | **Size** | **Biomarker(s)** | **AUC** | **Sensitivity** | **Specificity** |
| --- | --- | --- | --- | --- | --- |
| ANN | 5 | IL-5, CCL2, CD14^+^, CD3^+^, CCL13 | 0.621 ± *0.088* | 0.62 ± *0.15* | 0.60 ± *0.12* |
|  | 10 | + IFN-γ, IL-6, HNE, sIL-6R, IL-10 | 0.637 ± *0.200* | 0.63 ± *0.24* | 0.62 ± *0.24* |
| SVM | 5 | IL-17A, IFN-γ, IL-123p40, cell count, CXCL10 | 0.553 ± *0.100* | 0.49 ± *0.11* | 0.70 ± *0.18* |
|  | 10 | + MMP substr, CCL2, zymography, IL-1β, IL-2 | 0.691 ± *0.126* | 0.66 ± *0.29* | 0.58 ± *0.41* |
| RF | 5 | IL-17A, IL-12p40, IFN-γ, IL-1β, cell count | 0.711 ± *0.046* | 0.66 ± *0.10* | 0.68 ± *0.17* |
|  | 10 | + IL-2, Vγ9^+^, zymography, CXCL10, IL-16 | 0.744 ± *0.076* | 0.70 ± *0.13* | 0.69 ± *0.18* |
| ROC | 1 | IL-17A, cut-off: 5.7 pg/ml | 0.72 *(0.62–0.83)* | 0.77 | 0.61 |
|  | 1 | IL-12p40, cut-off: 81.7 pg/ml | 0.70 *(0.58–0.81)* | 0.75 | 0.67 |
|  | 1 | IFN-γ, cut-off: 58.9 pg/ml | 0.70 *(0.59–0.81)* | 0.49 | 0.86 |
|  | 1 | IL-1β, cut-off: 2.2 pg/ml | 0.61 *(0.48–0.75)* | 0.83 | 0.50 |
|  | 1 | Cell count, cut-off: 3.1 × 10^9^ cells | 0.66 *(0.53–0.79)* | 0.69 | 0.65 |

Shown are the biomarker combinations as selected by recursive feature elimination using RF, SVM and ANN models, listed in the order of the importance in the different models. The top 5 biomarkers from the RF model were also evaluated individually in conventional ROC analyses. AUC, specificity and sensitivity for machine learning model are shown as average and *SEM* values of the validation dataset after five rounds of re-sampling. Values for individual markers are shown as AUC with lower and higher confidence boundaries. Cut-off values were determined from the highest sum of sensitivity and specificity.
