# Supplementary material for: Machine-learning algorithms define pathogen-specific local immune fingerprints in peritoneal dialysis patients with bacterial infections
Source: Kidney Int. 2017 Jul;92(1):179–91. doi: 10.1016/j.kint.2017.01.017 (PMC5484022; doi:10.1016/j.kint.2017.01.017)
Supplement: Table S5A — Local biomarkers in patients presenting with acute peritonitis caused by streptococcal species (Streptococcus spp. and Enterococcus spp.) or with other episodes. [file mmc10.docx]

Supplementary Table S5A. Local biomarkers in patients presenting with acute peritonitis caused by streptococcal species (*Streptococcus spp.* and *Enterococcus spp.*) or with other episodes.

| Biomarker | **Streptococcal infections** | | **Other episodes** | | *p* |
| --- | --- | --- | --- | --- | --- |
|  | Mean | *SEM* | Mean | *SEM* |  |
| IL-1α (pg/ml) | 24.93 | *2.20* | 28.64 | *4.15* |  |
| IL-1β (pg/ml) | 53.40 | *16.01* | 30.11 | *9.35* | ** |
| IL-2 (pg/ml) | 7.44 | *1.77* | 11.18 | *2.82* |  |
| IL-4 (pg/ml) | 3.35 | *0.50* | 3.60 | *0.47* |  |
| IL-5 (pg/ml) | 1.29 | *0.23* | 2.50 | *0.48* |  |
| IL-6 (pg/ml) | 833.00 | *0.00* | 750.23 | *26.01* |  |
| IL-7 (pg/ml) | 2.45 | *0.13* | 4.24 | *0.53* | * |
| IL-10 (pg/ml) | 53.00 | *18.45* | 45.39 | *8.78* |  |
| IL-12p40 (pg/ml) | 173.45 | *38.61* | 207.46 | *55.56* |  |
| IL-12p70 (pg/ml) | 5.81 | *1.16* | 7.30 | *0.95* |  |
| IL-13 (pg/ml) | 19.22 | *2.00* | 21.18 | *2.63* |  |
| IL-15 (pg/ml) | 3.22 | *0.62* | 6.47 | *0.89* | 0.053 |
| IL-16 (pg/ml) | 460.42 | *99.28* | 487.21 | *76.55* |  |
| IL-17A (pg/ml) | 29.06 | *10.55* | 87.95 | *27.84* |  |
| IL-18 (pg/ml) | 40.22 | *8.93* | 103.48 | *23.77* |  |
| IL-22 (pg/ml) | 27.92 | *1.17* | 30.58 | *1.72* |  |
| sIL-6R (pg/ml) | 1445.44 | *167.56* | 1573.16 | *81.70* |  |
| IFN-γ (pg/ml) | 48.81 | *11.43* | 196.54 | *45.04* |  |
| TNF-α (pg/ml) | 144.22 | *37.70* | 76.34 | *14.67* | * |
| TNF-β (pg/ml) | 0.32 | *0.00* | 1.13 | *0.42* | ** |
| GM-CSF (pg/ml) | 1.33 | *0.22* | 2.10 | *0.35* |  |
| TGF-β (pg/ml) | 263.45 | *41.88* | 236.66 | *19.86* |  |
| VEGF (pg/ml) | 153.82 | *58.55* | 165.35 | *29.19* |  |
| CCL2 (pg/ml) | 560.00 | *0.00* | 464.34 | *18.78* | ** |
| CCL3 (pg/ml) | 404.41 | *107.53* | 287.41 | *48.48* |  |
| CCL4 (pg/ml) | 930.93 | *104.67* | 614.52 | *60.98* | * |
| CCL11 (pg/ml) | 1267.89 | *105.33* | 1030.43 | *68.82* |  |
| CCL13 (pg/ml) | 28.99 | *4.31* | 41.94 | *6.44* |  |
| CCL17 (pg/ml) | 80.42 | *11.15* | 127.20 | *27.35* |  |
| CCL22 (pg/ml) | 441.63 | *66.46* | 503.26 | *57.08* |  |
| CCL26 (pg/ml) | 72.13 | *10.64* | 69.25 | *7.98* |  |
| CXCL8 (pg/ml) | 5475.64 | *2157.63* | 3750.85 | *1488.50* | ** |
| CXCL10 (pg/ml) | 1954.01 | *268.16* | 1943.21 | *130.75* |  |
| MMP-8 total (ng/ml) | 28.08 | *3.22* | 22.79 | *1.92* |  |
| MMP substrate (ng/ml) | 20.45 | *2.98* | 16.60 | *1.64* |  |
| Human neutrophil elastase (ng/ml) | 15.69 | *4.77* | 11.86 | *2.30* |  |
| HNE substrate (ng/ml) | 2.16 | *0.20* | 1.82 | *0.13* | * |
| Zymography (arbitrary units) | 165.66 | *23.55* | 130.85 | *11.23* |  |
| Calprotectin (ng/ml) | 83.67 | *4.39* | 80.65 | *2.17* |  |
| Surfactant protein D (SPD) | 1.63 | *0.23* | 1.57 | *0.13* |  |
| Total cell count (× 10^9^ cells) | 13.38 | *5.29* | 6.29 | *0.95* |  |
| CD3^+^ (% of total) | 0.49 | *0.23* | 1.36 | *0.30* | * |
| CD14^+^ (% of total) | 7.10 | *1.51* | 13.21 | *1.56* | * |
| CD15^+^ (% of total) | 83.21 | *4.09* | 78.35 | *1.85* | * |
| CD4:CD8 ratio | 1.48 | *0.24* | 1.55 | *0.16* |  |
| CD4^+^ (% of T cells) | 48.58 | *2.95* | 48.50 | *2.09* |  |
| CD8^+^ (% of T cells) | 40.05 | *3.42* | 39.08 | *1.74* |  |
| Vγ9^+^ (% of T cells) | 2.16 | *0.43* | 3.25 | *0.42* |  |
| Vδ2^+^ (% of T cells) | 2.17 | *0.38* | 3.85 | *0.57* |  |

Differences between the two patient groups were considered statistically significant as indicated:
* *p*<0.05, ** *p*<0.01, *** *p*<0.001, based on two-tailed Mann-Whitney tests.
