# Supplementary material for: Machine-learning algorithms define pathogen-specific local immune fingerprints in peritoneal dialysis patients with bacterial infections
Source: Kidney Int. 2017 Jul;92(1):179–91. doi: 10.1016/j.kint.2017.01.017 (PMC5484022; doi:10.1016/j.kint.2017.01.017)
Supplement: Table S5B — Performance of local biomarkers in predicting infections caused by streptococcal species (Streptococcus spp. and Enterococcus spp.) against all other episodes of peritonitis. [file mmc11.docx]

Supplementary Table S5B. Performance of local biomarkers in predicting infections caused by streptococcal species (*Streptococcus spp.* and *Enterococcus spp.*) against all other episodes of peritonitis.

| **Model** | **Size** | **Biomarker(s)** | **AUC** | **Sensitivity** | **Specificity** | |
| --- | --- | --- | --- | --- | --- | --- |
| ANN | 5 | sIL-6R, Vγ9^+^, CCL11, cell count, CCL4 | 0.893 ± *0.042* | 0.76 ± *0.05* | 0.97 ± *0.04* |  |
|  | 10 | + CXCL10, CCL2, MMP substrate, IL-15,  zymography | 0.908 ± *0.039* | 0.79 ± *0.14* | 0.98 ± *0.03* |  |
| SVM | 5 | IL-1β, CD3^+^, CXCL8, CCL4, zymography | 0.971 ± *0.032* | 0.74 ± *0.42* | 0.95 ± *0.07* |  |
|  | 10 | + CD15^+^, CCL2, IL-7, IL-15, MMP substrate | 0.977 ± *0.034* | 0.60 ± *0.55* | 0.71 ± *0.44* |  |
| RF | 5 | IL-1β, MMP substrate, TNF-β, IL-15, zymography | 0.969 ± *0.046* | 0.84 ± *0.08* | 0.97 ± *0.04* |  |
|  | 10 | + IL-12p70, sIL-6R, CXCL8, IL-4, CD3^+^ | 0.989 ± *0.017* | 0.88 ± *0.11* | 0.97 ± *0.04* |  |
| ROC | 1 | IL-1β, cut-off: 2.9 pg/ml | 0.73 *(0.62–0.85)* | 1.00 | 0.49 |  |
|  | 1 | MMP substrate, cut-off: 25.9 pg/ml | 0.61 *(0.43–0.78)* | 0.50 | 0.89 |  |
|  | 1 | TNF-β, cut-off: *INF* | 0.32 *(0.26–0.38)* | 1.00 | 1.00 |  |
|  | 1 | IL-15, cut-off: 1.7 pg/ml | 0.66 *(0.51–0.80)* | 0.44 | 0.87 |  |
|  | 1 | Zymography, cut-off: 1.5 AU | 0.67 *(0.54–0.79)* | 0.81 | 0.57 |  |

Shown are the biomarker combinations as selected by recursive feature elimination using RF, SVM and ANN models, listed in the order of the importance in the different models. The top 5 biomarkers from the RF model were also evaluated individually in conventional ROC analyses. AUC, specificity and sensitivity for machine learning model are shown as average and *SEM* values of the validation dataset after five rounds of re-sampling. Values for individual markers are shown as AUC with lower and higher confidence boundaries. Cut-off values were determined from the highest sum of sensitivity and specificity.
