# Supplementary material for: Machine-learning algorithms define pathogen-specific local immune fingerprints in peritoneal dialysis patients with bacterial infections
Source: Kidney Int. 2017 Jul;92(1):179–91. doi: 10.1016/j.kint.2017.01.017 (PMC5484022; doi:10.1016/j.kint.2017.01.017)
Supplement: Table S6A — Local biomarkers in patients presenting with acute peritonitis caused by nonstreptococcal Gram-positive species (Staphylococcus aureus, coagulase-negative Staphylococcus spp., Corynebacterium spp.) or with other episodes. [file mmc12.docx]

Supplementary Table S6A. Local biomarkers in patients presenting with acute peritonitis caused by non-streptococcal Gram-positive species (*Staphylococcus aureus*, coagulase-negative *Staphylococcus spp.*, *Corynebacterium spp.*) or with other episodes.

| Biomarker | **Non-streptococcal Gram^+^ infections** | | **Other episodes** | | *p* |
| --- | --- | --- | --- | --- | --- |
|  | Mean | *SEM* | Mean | *SEM* |  |
| IL-1α (pg/ml) | 30.95 | *8.32* | 26.12 | *2.18* |  |
| IL-1β (pg/ml) | 32.62 | *18.18* | 35.78 | *7.47* |  |
| IL-2 (pg/ml) | 16.87 | *5.84* | 6.63 | *0.93* |  |
| IL-4 (pg/ml) | 3.94 | *0.71* | 3.33 | *0.46* |  |
| IL-5 (pg/ml) | 1.60 | *0.25* | 2.66 | *0.60* |  |
| IL-6 (pg/ml) | 799.28 | *25.86* | 746.46 | *30.11* |  |
| IL-7 (pg/ml) | 3.67 | *0.48* | 4.03 | *0.63* |  |
| IL-10 (pg/ml) | 40.15 | *12.03* | 50.86 | *10.40* |  |
| IL-12p40 (pg/ml) | 344.47 | *115.03* | 115.31 | *16.23* | * |
| IL-12p70 (pg/ml) | 8.24 | *1.51* | 6.28 | *0.90* |  |
| IL-13 (pg/ml) | 21.04 | *3.40* | 20.65 | *2.81* |  |
| IL-15 (pg/ml) | 7.30 | *1.42* | 4.97 | *0.81* | * |
| IL-16 (pg/ml) | 560.10 | *127.05* | 435.51 | *69.95* |  |
| IL-17A (pg/ml) | 162.71 | *56.96* | 25.26 | *6.57* | *** |
| IL-18 (pg/ml) | 131.87 | *47.17* | 67.47 | *12.83* |  |
| IL-22 (pg/ml) | 34.22 | *3.32* | 27.75 | *1.08* | 0.07 |
| sIL-6R (pg/ml) | 1584.54 | *123.91* | 1527.08 | *91.33* |  |
| IFN-γ (pg/ml) | 357.58 | *85.66* | 55.07 | *16.02* | *** |
| TNF-α (pg/ml) | 61.90 | *19.81* | 105.83 | *18.92* |  |
| TNF-β (pg/ml) | 1.89 | *0.89* | 0.43 | *0.04* | * |
| GM-CSF (pg/ml) | 2.54 | *0.68* | 1.60 | *0.22* |  |
| TGF-β (pg/ml) | 223.99 | *27.34* | 252.45 | *23.50* |  |
| VEGF (pg/ml) | 180.34 | *41.02* | 152.86 | *33.70* |  |
| CCL2 (pg/ml) | 498.86 | *21.88* | 473.19 | *21.45* |  |
| CCL3 (pg/ml) | 215.07 | *59.55* | 366.53 | *60.17* |  |
| CCL4 (pg/ml) | 623.80 | *87.68* | 706.35 | *70.24* |  |
| CCL11 (pg/ml) | 1142.02 | *101.37* | 1036.97 | *74.10* |  |
| CCL13 (pg/ml) | 38.13 | *6.37* | 40.23 | *7.58* |  |
| CCL17 (pg/ml) | 112.68 | *19.47* | 121.46 | *33.70* |  |
| CCL22 (pg/ml) | 539.66 | *75.79* | 462.60 | *61.59* |  |
| CCL26 (pg/ml) | 73.23 | *13.53* | 67.76 | *7.21* |  |
| CXCL8 (pg/ml) | 2237.11 | *1029.56* | 5183.97 | *1922.21* |  |
| CXCL10 (pg/ml) | 2318.19 | *161.43* | 1722.99 | *152.58* | ** |
| MMP-8 total (ng/ml) | 24.05 | *2.43* | 23.77 | *2.26* |  |
| MMP substrate (ng/ml) | 18.88 | *2.53* | 16.51 | *1.75* |  |
| Human neutrophil elastase (ng/ml) | 12.59 | *2.69* | 12.67 | *2.89* |  |
| HNE substrate (ng/ml) | 2.00 | *0.18* | 1.83 | *0.14* |  |
| Zymography (arbitrary units) | 157.37 | *17.14* | 126.52 | *12.54* |  |
| Calprotectin (ng/ml) | 80.96 | *3.01* | 81.45 | *2.54* |  |
| Surfactant protein D (SPD) | 1.75 | *0.23* | 1.49 | *0.11* |  |
| Total cell count (× 10^9^ cells) | 7.62 | *1.50* | 7.79 | *1.92* | 0.063 |
| CD3^+^ (% of total) | 1.83 | *0.54* | 0.80 | *0.22* | * |
| CD14^+^ (% of total) | 11.22 | *1.36* | 12.38 | *1.92* |  |
| CD15^+^ (% of total) | 80.83 | *2.07* | 78.48 | *2.41* |  |
| CD4:CD8 ratio | 1.77 | *0.30* | 1.41 | *0.13* |  |
| CD4^+^ (% of T cells) | 50.58 | *3.15* | 47.35 | *2.09* |  |
| CD8^+^ (% of T cells) | 38.16 | *2.93* | 39.93 | *1.77* |  |
| Vγ9^+^ (% of T cells) | 2.22 | *0.39* | 3.50 | *0.50* |  |
| Vδ2^+^ (% of T cells) | 3.27 | *0.84* | 3.61 | *0.55* |  |

Differences between the two patient groups were considered statistically significant as indicated:
* *p*<0.05, ** *p*<0.01, *** *p*<0.001, based on two-tailed Mann-Whitney tests.
