# Supplementary material for: Machine-learning algorithms define pathogen-specific local immune fingerprints in peritoneal dialysis patients with bacterial infections
Source: Kidney Int. 2017 Jul;92(1):179–91. doi: 10.1016/j.kint.2017.01.017 (PMC5484022; doi:10.1016/j.kint.2017.01.017)
Supplement: Table S6B — Performance of local biomarkers in predicting infections caused by nonstreptococcal Gram-positive species (Staphylococcus aureus, coagulase-negative Staphylococcus spp., Corynebacterium spp.) against all other episodes of peritonitis. [file mmc13.docx]

Supplementary Table S6B. Performance of local biomarkers in predicting infections caused by non-streptococcal Gram-positive species (*Staphylococcus aureus*, coagulase-negative *Staphylococcus spp.*, *Corynebacterium spp.*) against all other episodes of peritonitis.

| **Model** | **Size** | **Biomarker(s)** | **AUC** | **Sensitivity** | **Specificity** | |
| --- | --- | --- | --- | --- | --- | --- |
| ANN | 5 | CD3^+^, IL-17A, CCL3, IL-5, HNE | 0.712 ± *0.114* | 0.81 ± *0.16* | 0.60 ± *0.17* |  |
|  | 10 | + CD15^+^, IFN-γ, sIL-6R, CXCL10, CD14^+^ | 0.767 ± *0.052* | 0.77 ± *0.20* | 0.70 ± *0.20* |  |
| SVM | 5 | IL-17A, IFN-γ, IL-15, CXCL10, cell count | 0.796 ± *0.076* | 0.67 ± *0.18* | 0.79 ± *0.13* |  |
|  | 10 | + IL-12p40, TNF-α, CXCL8, Vδ2^+^, CCL22 | 0.738 ± *0.114* | 0.69 ± *0.16* | 0.83 ± *0.23* |  |
| RF | 5 | IL-17A, IFN-γ, IL-15, CCL3, cell count | 0.819 ± *0.039* | 0.73 ± *0.15* | 0.70 ± *0.22* |  |
|  | 10 | + TNF-β, IL-1β, TNF-α, IL-12p40, IL-13 | 0.887 ± *0.079* | 0.83 ± *0.16* | 0.75 ± *0.19* |  |
| ROC | 1 | IL-17A, cut-off: 16.2 pg/ml | 0.74 *(0.63–0.85)* | 0.61 | 0.75 |  |
|  | 1 | IFN-γ, cut-off: 58.9 pg/ml | 0.74 *(0.62–0.86)* | 0.61 | 0.83 |  |
|  | 1 | IL-15, cut-off: 3.0 pg/ml | 0.66 *(0.55–0.78)* | 0.58 | 0.74 |  |
|  | 1 | CCL3, cut-off: 68.9 pg/ml | 0.57 *(0.45–0.70)* | 0.65 | 0.58 |  |
|  | 1 | Cell count, cut-off: 2.0 × 10^9^ cells | 0.63 *(0.50–0.75)* | 0.48 | 0.86 |  |

Shown are the biomarker combinations as selected by recursive feature elimination using RF, SVM and ANN models, listed in the order of the importance in the different models. The top 5 biomarkers from the RF model were also evaluated individually in conventional ROC analyses. AUC, specificity and sensitivity for machine learning model are shown as average and *SEM* values of the validation dataset after five rounds of re-sampling. Values for individual markers are shown as AUC with lower and higher confidence boundaries. Cut-off values were determined from the highest sum of sensitivity and specificity.
