# Supplementary material for: Machine-learning algorithms define pathogen-specific local immune fingerprints in peritoneal dialysis patients with bacterial infections
Source: Kidney Int. 2017 Jul;92(1):179–91. doi: 10.1016/j.kint.2017.01.017 (PMC5484022; doi:10.1016/j.kint.2017.01.017)
Supplement: Table S7A — Local biomarkers in patients presenting with acute peritonitis caused by coagulase-negative staphylococci or with other episodes. [file mmc14.docx]

Supplementary Table S7A. Local biomarkers in patients presenting with acute peritonitis caused by coagulase-negative staphylococci or with other episodes.

| Biomarker | **CNS infections** | | **Other episodes** | | *p* |
| --- | --- | --- | --- | --- | --- |
|  | Mean | *SEM* | Mean | *SEM* |  |
| IL-1α (pg/ml) | 22.69 | *0.23* | 29.70 | *4.51* |  |
| IL-1β (pg/ml) | 19.01 | *7.57* | 39.88 | *10.60* |  |
| IL-2 (pg/ml) | 14.52 | *7.93* | 9.08 | *1.55* |  |
| IL-4 (pg/ml) | 3.76 | *0.91* | 3.49 | *0.43* |  |
| IL-5 (pg/ml) | 1.61 | *0.33* | 2.49 | *0.51* |  |
| IL-6 (pg/ml) | 783.22 | *37.96* | 760.42 | *25.54* |  |
| IL-7 (pg/ml) | 3.44 | *0.44* | 4.05 | *0.56* |  |
| IL-10 (pg/ml) | 46.68 | *17.12* | 46.92 | *8.91* |  |
| IL-12p40 (pg/ml) | 359.43 | *145.48* | 147.21 | *34.22* |  |
| IL-12p70 (pg/ml) | 7.48 | *1.69* | 6.85 | *0.91* |  |
| IL-13 (pg/ml) | 21.41 | *4.37* | 20.59 | *2.50* |  |
| IL-15 (pg/ml) | 8.57 | *2.02* | 4.92 | *0.69* | * |
| IL-16 (pg/ml) | 609.90 | *158.32* | 438.74 | *67.63* |  |
| IL-17A (pg/ml) | 160.58 | *81.16* | 48.15 | *11.88* | 0.073 |
| IL-18 (pg/ml) | 152.38 | *67.31* | 71.36 | *12.98* |  |
| IL-22 (pg/ml) | 31.56 | *3.80* | 29.59 | *1.42* |  |
| sIL-6R (pg/ml) | 1399.19 | *147.88* | 1599.13 | *83.94* |  |
| IFN-γ (pg/ml) | 290.94 | *96.28* | 126.44 | *36.23* |  |
| TNF-α (pg/ml) | 68.36 | *25.17* | 96.56 | *16.85* |  |
| TNF-β (pg/ml) | 2.46 | *1.30* | 0.47 | *0.04* |  |
| GM-CSF (pg/ml) | 2.64 | *0.95* | 1.72 | *0.22* |  |
| TGF-β (pg/ml) | 226.60 | *31.57* | 246.98 | *21.51* |  |
| VEGF (pg/ml) | 173.38 | *44.55* | 159.65 | *31.51* |  |
| CCL2 (pg/ml) | 496.00 | *27.80* | 478.30 | *18.88* |  |
| CCL3 (pg/ml) | 227.13 | *72.66* | 338.02 | *53.77* |  |
| CCL4 (pg/ml) | 641.60 | *106.55* | 687.01 | *64.15* |  |
| CCL11 (pg/ml) | 1116.97 | *120.00* | 1062.39 | *69.44* |  |
| CCL13 (pg/ml) | 33.27 | *6.98* | 41.53 | *6.67* |  |
| CCL17 (pg/ml) | 101.32 | *21.35* | 123.89 | *28.93* |  |
| CCL22 (pg/ml) | 532.67 | *87.85* | 477.40 | *56.86* |  |
| CCL26 (pg/ml) | 70.23 | *19.07* | 69.66 | *6.42* |  |
| CXCL8 (pg/ml) | 2479.88 | *1451.47* | 4626.44 | *1625.75* |  |
| CXCL10 (pg/ml) | 2153.23 | *222.62* | 1874.86 | *136.85* |  |
| MMP-8 total (ng/ml) | 22.22 | *3.11* | 24.41 | *1.98* |  |
| MMP substrate (ng/ml) | 17.64 | *3.02* | 17.31 | *1.65* |  |
| Human neutrophil elastase (ng/ml) | 12.79 | *3.63* | 12.59 | *2.48* |  |
| HNE substrate (ng/ml) | 1.95 | *0.23* | 1.87 | *0.12* |  |
| Zymography (arbitrary units) | 156.87 | *22.74* | 131.91 | *11.34* |  |
| Calprotectin (ng/ml) | 79.49 | *3.60* | 81.84 | *2.29* |  |
| Surfactant protein D (SPD) | 1.72 | *0.32* | 1.54 | *0.11* |  |
| Total cell count (× 10^9^ cells) | 7.81 | *2.11* | 7.70 | *1.63* |  |
| CD3^+^ (% of total) | 2.06 | *0.82* | 0.91 | *0.20* |  |
| CD14^+^ (% of total) | 11.02 | *1.59* | 12.24 | *1.64* |  |
| CD15^+^ (% of total) | 79.56 | *2.83* | 79.28 | *2.05* |  |
| CD4:CD8 ratio | 1.47 | *0.21* | 1.56 | *0.17* |  |
| CD4^+^ (% of T cells) | 49.24 | *3.28* | 48.32 | *2.06* |  |
| CD8^+^ (% of T cells) | 39.44 | *3.51* | 39.25 | *1.73* |  |
| Vγ9^+^ (% of T cells) | 2.09 | *0.46* | 3.31 | *0.43* |  |
| Vδ2^+^ (% of T cells) | 3.16 | *0.74* | 3.58 | *0.56* |  |

Differences between the two patient groups were considered statistically significant as indicated:
* *p*<0.05, ** *p*<0.01, *** *p*<0.001, based on two-tailed Mann-Whitney tests.
