# Supplementary material for: Machine-learning algorithms define pathogen-specific local immune fingerprints in peritoneal dialysis patients with bacterial infections
Source: Kidney Int. 2017 Jul;92(1):179–91. doi: 10.1016/j.kint.2017.01.017 (PMC5484022; doi:10.1016/j.kint.2017.01.017)
Supplement: Table S7B — Performance of local biomarkers in predicting infections caused by coagulase-negative staphylococci against all other episodes of peritonitis. [file mmc15.docx]

Supplementary Table S7B. Performance of local biomarkers in predicting infections caused by coagulase-negative staphylococci against all other episodes of peritonitis.

| **Model** | **Size** | **Biomarker(s)** | **AUC** | **Sensitivity** | **Specificity** | |
| --- | --- | --- | --- | --- | --- | --- |
| ANN | 5 | Calprotectin, HNE, SPD, zymography, CD8^+^ | 0.855 ± *0.022* | 0.97 ± *0.05* | 0.68 ± *0.05* |  |
|  | 10 | + MMP substrate, CD3^+^,IL-5, sIL-6R, CD14^+^ | 0.845 ± *0.117* | 0.95 ± *0.07* | 0.68 ± *0.17* |  |
| SVM | 5 | IL-15, HNE, IL-17A, CCL13, IL-12p40 | 0.961 ± *0.042* | 0.94 ± *0.07* | 0.97 ± *0.04* |  |
|  | 10 | + CXCL10, sIL-6R, IFN-γ, TNF-α, cell count | 0.962 ± *0.061* | 0.91 ± *0.14* | 1.00 ± *0.00* |  |
| RF | 5 | IL-15, IL-16, sIL-6R, cell count, MMP substrate | 0.961 ± *0.048* | 0.97 ± *0.07* | 0.82 ± *0.11* |  |
|  | 10 | + CCL17, CCL26, IL-17A, CD15^+^, IFN-γ | 0.958 ± *0.042* | 0.95 ± *0.07* | 0.79 ± *0.09* |  |
| ROC | 1 | IL-15, cut-off: 0.3 pg/ml | 0.68 *(0.56–0.80)* | 0.81 | 0.55 |  |
|  | 1 | IL-16, cut-off: 141.9 pg/ml | 0.60 *(0.46–0.73)* | 0.91 | 0.39 |  |
|  | 1 | sIL-6R, cut-off: 1354.0 pg/ml | 0.60 *(0.45–0.74)* | 0.67 | 0.68 |  |
|  | 1 | Cell count, cut-off: 2.0 × 10^9^ cells | 0.60 *(0.47–0.73)* | 0.90 | 0.43 |  |
|  | 1 | MMP substrate, cut-off: 8.7 ng/ml | 0.53 *(0.38–0.67)* | 0.90 | 0.32 |  |

Shown are the biomarker combinations as selected by recursive feature elimination using RF, SVM and ANN models, listed in the order of the importance in the different models. The top 5 biomarkers from the RF model were also evaluated individually in conventional ROC analyses. AUC, specificity and sensitivity for machine learning model are shown as average and *SEM* values of the validation dataset after five rounds of re-sampling. Values for individual markers are shown as AUC with lower and higher confidence boundaries. Cut-off values were determined from the highest sum of sensitivity and specificity.
