# Supplementary material for: Machine-learning algorithms define pathogen-specific local immune fingerprints in peritoneal dialysis patients with bacterial infections
Source: Kidney Int. 2017 Jul;92(1):179–91. doi: 10.1016/j.kint.2017.01.017 (PMC5484022; doi:10.1016/j.kint.2017.01.017)
Supplement: Table S8A — Local biomarkers in patients technique failure over the next 90 days or with other episodes. [file mmc16.docx]

Supplementary Table S8A. Local biomarkers in patients technique failure over the next 90 days or with other episodes.

| Biomarker | **Technique failure** | | **Other episodes** | | *p* |
| --- | --- | --- | --- | --- | --- |
|  | Mean | *SEM* | Mean | *SEM* |  |
| IL-1α (pg/ml) | 23.44 | *0.68* | 29.64 | *4.65* |  |
| IL-1β (pg/ml) | 24.72 | *7.95* | 38.39 | *10.90* |  |
| IL-2 (pg/ml) | 14.82 | *7.22* | 8.79 | *1.60* |  |
| IL-4 (pg/ml) | 3.01 | *0.47* | 3.76 | *0.51* |  |
| IL-5 (pg/ml) | 1.54 | *0.29* | 2.54 | *0.52* |  |
| IL-6 (pg/ml) | 800.29 | *25.40* | 753.11 | *27.71* |  |
| IL-7 (pg/ml) | 3.29 | *0.39* | 4.13 | *0.58* |  |
| IL-10 (pg/ml) | 52.79 | *10.98* | 44.59 | *10.11* |  |
| IL-12p40 (pg/ml) | 186.38 | *70.54* | 206.47 | *57.00* |  |
| IL-12p70 (pg/ml) | 6.30 | *0.99* | 7.28 | *1.04* |  |
| IL-13 (pg/ml) | 19.73 | *2.97* | 21.21 | *2.77* |  |
| IL-15 (pg/ml) | 7.25 | *1.43* | 5.30 | *0.86* | * |
| IL-16 (pg/ml) | 548.85 | *119.57* | 456.43 | *76.86* |  |
| IL-17A (pg/ml) | 83.95 | *29.64* | 73.78 | *29.36* |  |
| IL-18 (pg/ml) | 92.82 | *25.14* | 91.13 | *25.63* |  |
| IL-22 (pg/ml) | 32.54 | *3.29* | 29.10 | *1.48* |  |
| sIL-6R (pg/ml) | 1762.47 | *118.45* | 1466.53 | *88.79* | 0.078 |
| IFN-γ (pg/ml) | 278.90 | *98.44* | 125.57 | *33.56* |  |
| TNF-α (pg/ml) | 74.80 | *23.89* | 95.03 | *17.28* |  |
| TNF-β (pg/ml) | 2.14 | *1.19* | 0.52 | *0.06* |  |
| GM-CSF (pg/ml) | 2.34 | *0.88* | 1.80 | *0.22* |  |
| TGF-β (pg/ml) | 274.56 | *28.86* | 229.28 | *22.04* |  |
| VEGF (pg/ml) | 174.82 | *51.75* | 158.64 | *30.22* |  |
| CCL2 (pg/ml) | 506.64 | *24.91* | 473.63 | *19.50* |  |
| CCL3 (pg/ml) | 369.85 | *96.11* | 287.00 | *49.16* |  |
| CCL4 (pg/ml) | 745.66 | *104.21* | 648.63 | *64.47* |  |
| CCL11 (pg/ml) | 1043.05 | *114.03* | 1088.91 | *70.75* |  |
| CCL13 (pg/ml) | 25.75 | *3.76* | 44.69 | *7.07* |  |
| CCL17 (pg/ml) | 94.22 | *19.23* | 127.37 | *29.88* |  |
| CCL22 (pg/ml) | 488.45 | *77.62* | 492.51 | *59.35* |  |
| CCL26 (pg/ml) | 62.65 | *8.14* | 72.54 | *8.79* |  |
| CXCL8 (pg/ml) | 3780.77 | *1568.84* | 4199.32 | *1655.75* |  |
| CXCL10 (pg/ml) | 1909.19 | *238.68* | 1959.14 | *134.45* |  |
| MMP-8 total (ng/ml) | 27.95 | *2.49* | 22.47 | *2.06* |  |
| MMP substrate (ng/ml) | 19.77 | *3.28* | 16.57 | *1.58* |  |
| Human neutrophil elastase (ng/ml) | 15.30 | *4.14* | 11.73 | *2.39* |  |
| HNE substrate (ng/ml) | 1.91 | *0.18* | 1.89 | *0.13* |  |
| Zymography (arbitrary units) | 153.66 | *20.98* | 132.58 | *11.69* |  |
| Calprotectin (ng/ml) | 85.65 | *2.69* | 79.76 | *2.41* |  |
| Surfactant protein D (SPD) | 1.49 | *0.19* | 1.62 | *0.14* |  |
| Total cell count (× 10^9^ cells) | 11.86 | *3.83* | 6.23 | *1.13* | * |
| CD3^+^ (% of total) | 1.89 | *0.75* | 0.93 | *0.21* |  |
| CD14^+^ (% of total) | 10.81 | *1.89* | 12.35 | *1.64* |  |
| CD15^+^ (% of total) | 79.22 | *3.01* | 79.39 | *2.05* |  |
| CD4:CD8 ratio | 1.14 | *0.10* | 1.68 | *0.18* |  |
| CD4^+^ (% of T cells) | 44.79 | *2.57* | 49.83 | *2.17* |  |
| CD8^+^ (% of T cells) | 42.34 | *2.43* | 38.21 | *1.89* |  |
| Vγ9^+^ (% of T cells) | 2.97 | *0.59* | 3.05 | *0.43* |  |
| Vδ2^+^ (% of T cells) | 3.23 | *0.67* | 3.57 | *0.58* |  |

Differences between the two patient groups were considered statistically significant as indicated:
* *p*<0.05, ** *p*<0.01, *** *p*<0.001, based on two-tailed Mann-Whitney tests.
