# Supplementary material for: Machine-learning algorithms define pathogen-specific local immune fingerprints in peritoneal dialysis patients with bacterial infections
Source: Kidney Int. 2017 Jul;92(1):179–91. doi: 10.1016/j.kint.2017.01.017 (PMC5484022; doi:10.1016/j.kint.2017.01.017)
Supplement: Table S8B — Performance of local biomarkers in predicting technique failure over the next 90 days against all other episodes of peritonitis. [file mmc17.docx]

Supplementary Table S8B. Performance of local biomarkers in predicting technique failure over the next 90 days against all other episodes of peritonitis.

| **Model** | **Size** | **Biomarker(s)** | **AUC** | **Sensitivity** | **Specificity** | |
| --- | --- | --- | --- | --- | --- | --- |
| ANN | 5 | sIL-6R, HNE substrate, SPD, TGF-β, HNE | 0.848 ± *0.043* | 0.93 ± *0.07* | 0.67 ± *0.13* |  |
|  | 10 | + cell count, CD3^+^, calprotectin, MMP-8,   TNF-α | 0.872 ± *0.074* | 0.95 ± *0.05* | 0.70 ± *0.15* |  |
| SVM | 5 | TGF-β, HNE, HNE substrate, IL-15, cell count | 0.900 ± *0.089* | 0.78 ± *0.14* | 0.93 ± *0.09* |  |
|  | 10 | + CCL13, MMP-8, sIL-6R, CD3^+^, CD15^+^ | 0.938 ± *0.065* | 0.90 ± *0.09* | 0.95 ± *0.07* |  |
| RF | 5 | Calprotectin, MMP-8, sIL-6R, CD4:CD8, TGF-β | 0.911 ± *0.060* | 0.92 ± *0.10* | 0.73 ± *0.20* |  |
|  | 10 | + IL-15, CCL13, CCL22, CD14^+^, zymography | 0.938 ± *0.082* | 0.93 ± *0.11* | 0.82 ± *0.16* |  |
| ROC | 1 | Calprotectin, cut-off: 70.1 ng/ml | 0.60 *(0.42–0.69)* | 0.29 | 1.00 |  |
|  | 1 | MMP-8, cut-off: 20.4 ng/ml | 0.61 *(0.48–0.74)* | 0.50 | 0.80 |  |
|  | 1 | sIL-6R, cut-off: 1143.1 pg/ml | 0.63 *(0.50–0.75)* | 0.37 | 0.96 |  |
|  | 1 | CD4:CD8 ratio, cut-off: 1.84 | 0.59 *(0.46–0.73)* | 0.37 | 0.94 |  |
|  | 1 | TGF-β, cut-off: 151.3 ng/ml | 0.61 *(0.48–0.74)* | 0.42 | 0.87 |  |

Shown are the biomarker combinations as selected by recursive feature elimination using RF, SVM and ANN models, listed in the order of the importance in the different models. The top 5 biomarkers from the RF model were also evaluated individually in conventional ROC analyses. AUC, specificity and sensitivity for machine learning model are shown as average and *SEM* values of the validation dataset after five rounds of re-sampling. Values for individual markers are shown as AUC with lower and higher confidence boundaries. Cut-off values were determined from the highest sum of sensitivity and specificity.
