# Supplementary material for: Machine-learning algorithms define pathogen-specific local immune fingerprints in peritoneal dialysis patients with bacterial infections
Source: Kidney Int. 2017 Jul;92(1):179–91. doi: 10.1016/j.kint.2017.01.017 (PMC5484022; doi:10.1016/j.kint.2017.01.017)
Supplement: Table S9 — Proportion of missing values. [file mmc18.docx]

Supplementary Table S9. Proportion of missing values.

| Biomarker | **% of missing values** |
| --- | --- |
| IL-18 | 3.6 |
| Total cell count | 4.8 |
| IL-22 | 6.0 |
| MMP substrate | 6.0 |
| HNE substrate | 6.0 |
| Zym | 6.0 |
| MMP8 Total | 6.0 |
| Human neutrophil elastase (HNE) | 6.0 |
| Calprotectin | 6.0 |
| Surfactant protein D (SPD) | 6.0 |
| CD3^+^ (% of total) | 12.1 |
| CD14^+^ (% of total) | 12.1 |
| CD15^+^ (% of total) | 12.1 |
| Vγ9^+^ (% of T cells) | 12.1 |
| CD4:CD8 ratio | 16.9 |
| CD4^+^ (% of T cells) | 16.9 |
| CD8^+^ (% of T cells) | 16.9 |
| Vδ2^+^ (% of T cells) | 16.9 |
